# Supplementary material for: Confound-leakage: confound removal in machine learning leads to leakage
Source: Gigascience. 2023 Sep 30;12:giad071. doi: 10.1093/gigascience/giad071 (PMC10541796; doi:10.1093/gigascience/giad071)
Supplement: giad071_GIGA-D-23-00004_Revision_1 [file giad071_giga-d-23-00004_revision_1.pdf]

GigaScience

Confound-leakage: Confound Removal in Machine Learning Leads to Leakage

--Manuscript Draft--

**Manuscript Number:**	GIGA-D-23-00004R1
**Full Title:**	Confound-leakage: Confound Removal in Machine Learning Leads to Leakage
**Article Type:**	Research
**Funding Information:**	Helmholtz Artificial Intelligence Cooperation Unit (ZT-I-PF-5-078) — Dr. Kaustubh R. Patil<br>Helmholtz Portfolio Thema 'Supercomputing and Modeling for the Human Brain' — Prof. Simon B. Eickhoff<br>Deutsche Forschungsgemeinschaft (Project-ID 431549029 – SFB 1451 project B05) — Prof. Simon B. Eickhoff
**Abstract:**	Background<br>Machine learning (ML) approaches are a crucial component ofmodern data analysis in many fields including epidemiology and medicine. Nonlinear MLmethods often achieve accurate predictions, for instance in personalizedmedicine, as they are capable of modeling complex relationships between features and the target. Problematically, MLmodels and their predictions can be biased by confounding information present in the features. To remove this spurious signal, researchers often employ featurewise linear confound regression (CR).While this is considered a standard approach for dealing with confounding, possible pitfalls of using CR in ML pipelines are not fully understood.<br>Results<br>We provide new evidence that, contrary to general expectations, linear confound regression can increase the risk of confounding when combined with nonlinear ML approaches. Using a simple framework that uses the target as a confound, we show that information leaked via CR can increase null ormoderate effects to near-perfect prediction. By shuffling the features we provide evidence that this increase is indeed due to confound-leakage and not due to revealing of information. We then demonstrate the danger of confound-leakage in a real-world clinical application where the accuracy of predicting attention deficit hyperactivity disorder is overestimated using speech-derived features when using depression as a confound.<br>Conclusions<br>Mishandling or even amplifying confounding effects when building MLmodels due to confound-leakage, as shown, can lead to untrustworthy, biased, and unfair predictions. Our expose of the confound-leakage pitfall and provided guidelines for dealing with it can help createmore robust and trustworthy MLmodels.
**Corresponding Author:**	Kaustubh R. Patil<br>Forschungszentrum Jülich: Forschungszentrum Julich GmbH<br>Jülich, NRW GERMANY
**Corresponding Author Secondary Information:**	
**Corresponding Author's Institution:**	Forschungszentrum Jülich: Forschungszentrum Julich GmbH
**Corresponding Author's Secondary Institution:**	
**First Author:**	Sami Hamdan
**First Author Secondary Information:**	
**Order of Authors:**	Sami Hamdan<br>Bradley C. Love<br>Georg G. von Polier<br>Susanne Weis

Powered by Editorial Manager® and ProduXion Manager® from Aries Systems Corporation

	Holger Schwender
	Simon B. Eickhoff
	Kaustubh R. Patil
**Order of Authors Secondary Information:**	
**Response to Reviewers:**	The point-by-point response was uploaded as a "Personal cover" PDF file.<br>Please note that there are two "Personal cover" files, one the actual cover letter and the other contains response to reviewers' comments.<br><br>We thank the reviewers for their insightful and detailed comments and the oppourtunity to revise our manuscript.<br>We have addressed all the comments and belive that the manuscript has improved considerably and the new analyses support the main finding of "confound leakage".
**Additional Information:**	
**Question**	**Response**
Are you submitting this manuscript to a special series or article collection?	No
**Experimental design and statistics**<br><br>Full details of the experimental design and statistical methods used should be given in the Methods section, as detailed in our Minimum Standards Reporting Checklist. Information essential to interpreting the data presented should be made available in the figure legends.<br><br>Have you included all the information requested in your manuscript?	Yes
**Resources**<br><br>A description of all resources used, including antibodies, cell lines, animals and software tools, with enough information to allow them to be uniquely identified, should be included in the Methods section. Authors are strongly encouraged to cite Research Resource Identifiers (RRIDs) for antibodies, model organisms and tools, where possible.<br><br>Have you included the information requested as detailed in our Minimum Standards Reporting Checklist?	Yes
**Availability of data and materials**	No

Powered by Editorial Manager® and ProduXion Manager® from Aries Systems Corporation

All datasets and code on which the conclusions of the paper rely must be either included in your submission or deposited in publicly available repositories (where available and ethically appropriate), referencing such data using a unique identifier in the references and in the “Availability of Data and Materials” section of your manuscript.<br><br>Have you have met the above requirement as detailed in our Minimum Standards Reporting Checklist?	
If not, please give reasons for any omissions below.<br><br>as follow-up to "**Availability of data and materials**<br><br>All datasets and code on which the conclusions of the paper rely must be either included in your submission or deposited in publicly available repositories (where available and ethically appropriate), referencing such data using a unique identifier in the references and in the “Availability of Data and Materials” section of your manuscript.<br><br>Have you have met the above requirement as detailed in our Minimum Standards Reporting Checklist?<br><br>"	The ADHD-related data is not availably publically. This sensitive data is available from PeakPro-filing GmbH with certain restrictions. Restrictions apply to the availability of the data, which were used under licence for this study. Please contact Jörg Langner the co-founder and CTO of PeakProfil-ing GmbH with requests.

Powered by Editorial Manager® and ProduXion Manager® from Aries Systems Corporation

Manuscript
Click here to access/download;Manuscript;Pitfalls_of_Confound_Regression

GigaScience, 2023, 1–10

doi: xx.xxxx/xxxx
Manuscript in Preparation
Paper

PAPER

Confound-leakage: Confound Removal in Machine Learning Leads to Leakage

Sami Hamdan[1,2], Bradley C. Love[3,4,5], Georg G. von Polier[1,6,7], Susanne Weis[1,2], Holger Schwender[8], Simon B. Eickhoff[1,2], and Kaustubh R. Patil[1,2,*]

[1]Institute of Neuroscience and Medicine, Brain and Behaviour (INM-7), Forschungszentrum Jülich, Jülich, Germany and [2] Institute of Systems Neuroscience, Medical Faculty, Heinrich-Heine University Düsseldorf, Düsseldorf, Germany and [3] Department of Experimental Psychology, University College London, London, UK and [4] The Alan Turing Institute, London, UK and [5] European Lab for Learning & Intelligent Systems (ELLIS) and [6] Department of Child and Adolescent Psychiatry, Psychosomatics and Psychotherapy, University Hospital Frankfurt, Frankfurt, Germany and [7] Department of Child and Adolescent Psychiatry, Psychosomatics and Psychotherapy, RWTH Aachen University, Aachen, Germany and [8] Institute of Mathematics, Heinrich-Heine University Düsseldorf, Düsseldorf, Germany

[*]k.patil@fz-juelich.de

Abstract

Background

Machine learning (ML) approaches are a crucial component of modern data analysis in many fields including epidemiology and medicine. Nonlinear ML methods often achieve accurate predictions, for instance in personalized medicine, as they are capable of modeling complex relationships between features and the target. Problematically, ML models and their predictions can be biased by confounding information present in the features. To remove this spurious signal, researchers often employ featurewise linear confound regression (CR). While this is considered a standard approach for dealing with confounding, possible pitfalls of using CR in ML pipelines are not fully understood.

Results

We provide new evidence that, contrary to general expectations, linear confound regression can increase the risk of confounding when combined with nonlinear ML approaches. Using a simple framework that uses the target as a confound, we show that information leaked via CR can increase null or moderate effects to near-perfect prediction. By shuffling the features we provide evidence that this increase is indeed due to confound-leakage and not due to revealing of information. We then demonstrate the danger of confound-leakage in a real-world clinical application where the accuracy of predicting attention deficit hyperactivity disorder is overestimated using speech-derived features when using depression as a confound.

Conclusions

Mishandling or even amplifying confounding effects when building ML models due to confound-leakage, as shown, can lead to untrustworthy, biased, and unfair predictions. Our expose of the confound-leakage pitfall and provided guidelines for dealing with it can help create more robust and trustworthy ML models.

Key words: confounding; data-leakage; machine-learning; clinical applications

Key Points

- Confound removal is essential for building insightful and trustworthy ML models
- Confound removal can increase performance when combined with nonlinear ML
- This can be due to confound information leaking into the features
- Possible reasons are skewed feature distributions and feature of limited precision
- Confound removal should be applied with utmost care in combination with nonlinear ML

Introduction

Machine learning (ML) approaches have revolutionized biomedical data analysis by providing powerful tools, especially nonlinear models, that can model complex feature-target relationships [1, 2]. However, the very power these nonlinear models bring to data analysis also lead to new challenges. Specifically, as we will detail, when a standard confound removal approach is paired with nonlinear models, new and surprising issues arise as the unintended is discovered and misinterpreted as a true effect.

Imagine building a diagnostic classifier for attention deficit hyperactivity disorder (ADHD) based on speech patterns. This will be a useful clinical tool aiding objective diagnosis [3]. However, like most disorders, ADHD has comorbidity, for instance with depression. Ideally, an ADHD diagnostic classifier should only rely upon characteristics of ADHD and ignore that of depression. This is an example of confounding, where it is desirable that the confound depression is disregarded by the classifier. Another example of confounding is the effect of ageing and neurodegenerative diseases on the brain. In a study to build a neuroimaging-based diagnostic classifier, the non-pathological ageing signal is confounding [4]. Confounding is ubiquitous and further examples include batch effects in genomics [5, 6, 7], scanner effects in neuroimaging [8], patient and process information in radiographs [9], and group differences like naturally different brain sizes in investigation of brain-size-independent sex differences [10, 11]. Ignoring confounding effects in an ML application can render predictions untrustworthy and insights questionable [12] as this information can be exploited by learning algorithms [13] leading to spurious feature-target relationships [14], e.g., classification based on depression instead of ADHD or age instead of neuronal pathology. The benefits of big data in ML applications are obvious, especially when modeling weak relationships, but big data also leads to an increased risk of inducing confounded models [4, 15, 16, 11]. Confounding, thus, is a crucial concern and if not properly treated can threaten real-world applicability of ML.

When confounding masks the true feature-target relationship, its removal can clean the signal of interest leading to higher generalizability, e.g. removal of batch effects in genomics [7]. On the other hand, when confounding introduces artefactual relationships the same procedure can reduce prediction accuracy [17, 18]. In either case, removing or adjusting for confounding effects is crucial for obtaining unbiased results, as otherwise a ML model might mostly rely on confounds, rendering signals of interest redundant. Two methods for treating confounding are commonly employed in data analysis with the goal of building an accurate ML model that is not biased by the confounding information. Data can be stratified based on the confounding variables, but it may introduce confounding information [19], falsely increase test-set performance by removing harder to classify data points [20], and can result in excessive data loss. As confounds share variation -usually presumed linear variance- with both the target and the features, another common method is confound regression (CR) which removes the confounding variance, also called confounded signal, from each feature separately using a linear regression model [20, 4]. The resulting residualized features are considered confound-free and are used for subsequent analysis. CR has become the default method to counter confounding in observational studies, including in ML applications [20, 21, 16]. Typically, a two-step CR-ML workflow is constructed while avoiding risks associated with typical data-leakage by applying CR in a cross-validation-consistent manner [20, 22]. It is important to note that, we use a practitioner-oriented operational definition of confounds as a set of variables suspected to share an unwanted effect with both the features and target, which does not imply causality as in more formal definitions [23].

A CR-ML workflow typically attenuates prediction performance as it removes variance from the features that is informative of the target. If an increase in performance is observed after CR, it can be explained by either (1) *information-reveal*: CR reveals information that was masked by confounding or (2) *confound-leakage*: leakage of confounding information into the features. In the case of information-reveal, CR could suppress linear confounding or noise in turn enhancing the underlying (non-)linear signal and making learning easier for a suitable ML algorithm [13]. This would be a positive effect similar to removing simple shortcuts in the data [24, 25]. If this is the case then the resulting CR-ML workflow would be a valuable for modeling non-linear relationships. Alternatively, as CR is a univariate operation applied to each feature, multivariate confounding (across features) could be revealed, which could help prediction albeit undesirably. On the other hand, confound-leakage would be an even more worrisome outcome as it would leak confounding information into the features instead of removing it. Confound-leakage would be detrimental to the validity and interpretability of the ensuing CR-ML workflow and in some cases could lead to dangerous outcomes. CR has been reported to induce biases into statistical workflows, albeit not incorporating ML, leading to incorrectly inflated group differences inference in combined batch effects removal and group difference analysis [26]. It is important to note that CR is not without other pitfalls, for instance it might fail to completely remove confounding information [21, 27]. Still, CR is considered the de facto method, and therefore analyzing the hitherto unknown pitfall of leaking confounding information through CR is helpful. Furthermore, there were speculations of confound-leakage in ML workflows [18], it has not yet been systematically shown, analyzed nor explained.

To disentangle the two possible explanations of performance increase after CR, we systematically analyzed the two-step CR-ML workflow. For analysis purposes and to gain detailed knowledge, we propose a framework that uses the target as a confound (TaCo), in which we use a single confound that is the target. As a confound needs to share variation with both the target and the feature, any possible confound must share all confounded signal with the target. Hence, the target can be seen as a “superconfound” subsuming all possible confounding effects. Although it is unlikely to encounter a

Compiled on: May 26, 2023.
Draft manuscript prepared by the author.

confound equal to the target in real applications, TaCo provides a framework for systematic evaluation. It should be noted that real confounds will fall on the continuum from weak (low confounded signal) to strong (TaCo) depending on their degree of similarity with the target. Indeed, as we show, the TaCo framework reveals strong effects where the prediction accuracy is boosted from moderate to perfect as well as weaker effects for confounds weakly correlated with the target. A previous work has used TaCo for evaluating the validity and reliability of confound adjustment methods [21].

To this end, we performed extensive empirical analyses on several benchmark datasets providing strong evidence for confound-leakage. First, we showcase confound-leakage in a walk-through analyses. Then using the TaCo framework we systematically answer whether the improvement in prediction performance after CR is due to leakage. For this, we used benchmark datasets as well as several conceptually simple simulations covering both classification and regression problems. Finally, with a clinically-relevant task of ADHD diagnosis using speech-related features with depression as a confound, we demonstrate misleading impact of confound-leakage.

Results

Walk-through analysis

The goal of this section is to introduce readers to our analysis approach with intuitive examples. We show one exemplary case of TaCo removal for a binary classification task and a CR scenario with a weaker confound in a regression task. In both cases, we randomly split the data into 70% train and 30% test parts. The CR and prediction models were learned on the training data and the results are reported on the test split. We will show that, confound-leakage can be concluded if performance using shuffled features after CR ($\tilde{X}_{CR}$).

TaCo removal for binary classification

We analyzed the "bank investment" data to predict whether a customer will subscribe to term deposit given their financial and socio-economic information. We used a decision tree (DT) with limited maximum depth of two for visualization ease. This example is meant to demonstrate key aspects of our proposed analyses (Fig. 1).

TaCo removal showed a much higher area under the curve for the receiver operating characteristic curve (AUCROC) of 0.98 compared to the baseline AUCROC of 0.75 without CR. Still, the TaCo removed features were highly similar to the original features (median Pearson's correlation: 0.99, Fig. 1 a-b). The two ensuing DTs were, however, completely different and relied on different features. Notably, these drastic differences were induced by minute feature alterations after CR that are hardly detectable by humans but are effectively captured by DT (Fig. 1 c-d). Such performance increase can be either due to revealed information or confound-leakage. Therefore, we sought to gain evidence to distinguish between these two scenarios using two complementary measurements: 1) destroying the relationship between features and target, and 2) use of confound-predicted features.

To destroy the feature-target relation we shuffled each feature before CR ($\tilde{X}$) to create $\tilde{X}_{CR}$ and repeated the analysis. As there should be no predictive information in the shuffled features, the only explanation for above chance-level performance is CR leaking information into the confound-removed features X_{CR}, i.e. confound-leakage. We applied the shuffling procedure to a train-test split in this walk through analysis. But it should be noted that when combined with a (nested) cross-validation and Bayesian ROPE approach, this procedure can be used to compare models similarly as a permutation test (see section Shuffling the features and permutation testing). We observed chance-level performance without CR (AUCROC = 0.48) for the shuffled features. However, a performance increase after TaCo removal was observed (AUCROC = 0.99). This analysis shows that performance increase after TaCo removal with shuffled features indicate the possibility of confound-leakage.

Confound removal for regression

As an example of a weaker confound on a regression task, we simulated a binary confound and then sampled a feature from different distributions for each confound value (confound equal to 0 or 1). Then we added the confound to a normally distributed target ($M = 0$ and $SD = 0.50$,Fig. 1 e-f). This creates a clear confounding situation, where the confound affects both the feature (Point-biserial correlation = 0.71, $p < 0.01$) and the target (Point-biserial correlation = 0.71, $p < 0.01$) and thus leads to a spurious relationship between the feature and the target (Pearson's correlation = 0.51, $p < 0.01$). Following the same procedure as in the previous example, we observed increased performance after CR using a DT with limited depth of two (R^2 using $X = 0.29$, $X_{CR} = 0.42$). As in this simulated data only a spurious relation (via confound) exists between the feature and target, it is safe to assume that an increased performance after CR is due to confound-leakage. Furthermore, we found a probable mechanism behind this confound-leakage to be the distribution of the features conditioned on the confound. More precisely, CR shifts the feature values for confound = 1 in between most feature values for the confound = 0 (Fig. 1 e). This leaks the confounding information into the feature instead of removing it (Fig. 1 f). The shuffled features, however, were not sensitive to confound-leakage ($X = 0$,$\tilde{X} = -0.01$), which is expected considering the probable cause for such leakage depends on the joint distribution of the confound and the feature. When shuffling the features within each confound category to preserve the joint distribution, we observed an increase in performance after CR ($M = 0.29$ before to $M = 0.42$). This result indicates that shuffling the features might not be always sensitive to confound-leakage. We, nevertheless, use independently shuffled features in our analysis for practicality, particularly in the context of continuous or multiple confounding factors.

Analyses of benchmark data

TaCo removal increases performance of nonlinear methods

Our systematic and CV-consistent analysis comprised comparison between TaCo removal pipelines and no-CR pipelines on 10 UC Irvine (UCI) datasets . TaCo removal led to a meaningful increase in out-of-sample scoring using all tested non-linear models, RF (7/10 datasets), DT (8/10) SVM with RBF kernel (5/10) and MLP (7/10) (Fig. 2, Supplementary Fig. S1). This suggests that confound-leakage is a risk associated with the usage of a CR-ML pipeline with non-linear ML models. Furthermore, this suggests that the DT-based algorithms (DT and RF) are most susceptible to showing increased performance.

CR using weaker confounds also increases performance

As the target is the strongest possible confound, TaCo represents an extreme case. To test whether the potential leakage we found with TaCo extends to CR in general, using the UCI datasets we simulated confounds related to the target at different strengths measured by Pearson's correlation ranging 0.2 – 0.8. Depending on the dataset, different amounts of correlated confounds led to leakage after CR. We observed potential confound-leakage for 5 of the 10 datasets with at least one of the confound-target strengths. As expected, a higher target-confound correlation led to more leakage, i.e., higher performance after CR (Fig. 2 C).

Increased performance after TaCo removal is due to confound-leakage

As described in the walk-through analysis (see TaCo removal for binary classification), we measure the performance after first shuffling the features to evaluate whether the increased performance after TaCo removal/CR is due to information reveal or confound-

leakage. After shuffling the features, both pipelines, no-CR and TaCo removal, should perform close to chance-level if the improved performance is due to revealed information. Indeed, the no-CR pipeline performed close to the chance level, while TaCo removal pipeline increased the performance (Fig. 2 TaCo CR Shuffled). As there should be no predictive information in the shuffled features, above chance-level performance could only be obtained if the CR leaks information. Thus this result provides strong evidence in-favor of the confound-leakage.

For the simulated weaker confounds these results were less strong, still we found 5/10 datasets where X_{CR}, 9/10 where $\tilde{X}_{CR}$ performed above chance-level.

Possible mechanisms for confound-leakage

As a multitude of mechanisms could lead to confound-leakage, exhaustively identifying all possible mechanisms is out of the scope of this paper. Rather we want to highlight two possible mechanisms leading to confound-leakage inspired by the walk-through analyses: 1) Confound-leakage due to continuous features deviating from normal distributions (see Confound removal for regression) 2) Confound-leakage due to unbalanced features of limited precision (see TaCo removal for binary classification). Both mechanisms could be summarized under the umbrella of (small) differences of the conditional distributions of features given the confound inside of CV-folds.

As DT-based models are very popular ML algorithms [28] and seem to be most susceptible to the described problems (see TaCo removal increases performance of nonlinear methods) we will focus on them in our simulations to decrease the complexity of our results. Furthermore, we will use a DT whenever there is only one features and RF when there are multiple features.

Confound-leakage due to deviation from normal distributions

Consider simulating a standard normal feature not informative of a binary target. Then consider adding a smaller distribution around opposing extreme values separately for each class of a binary target (Fig. 3 a). The resulting feature only differs systematically w.r.t. the classes at the extreme values. As CR with a binary confound is equivalent to subtracting the mean for each confounding group from the respective feature, this operation is now biased towards the extreme parts of the feature distribution. Consequently, X_{CR} exposes confounding information in terms of decrease in the overlap of the feature distributions conditioned on the confound (Fig. 3 a-b). In other words, confounding information leaked via CR in turn increasing the prediction performance (AUROC from 0.51 before to 0.58 after TaCo removal). To show that the increased performance is not only due to better prediction of extreme values, we also tested the same model on a test set without the extreme values. The results were in line with previous observations, as the AUROC improved from 0.48 before to 0.57 after CR.

We also observed higher performance after similar decreased overlap due to TaCo removal in a simplified version of the "house pricing" UCI benchmark dataset (3 c-d), providing real world evidence for this phenomena.

Lastly, we investigated whether such effects could also occur when randomly sampling non-normal distributed features instead of carefully constructing the features conditioned on the confound. To this end, we sampled an increasing number of features (1 to 100) either using a random normal or skewed (χ^2, df = 3) distribution independent of a normally distributed target.

Using RF, we observed increased performance after TaCo removal with skewed features but not with normally distributed features, e.g. R^2 of M = 0.23 with SD = 0.06 compared to R^2 of M = −0.04 with SD = 0.04, respectively with 100 features. Importantly, this effect increased with the number of features (Fig 5). To further illustrate this point, we performed another simulation depicting a typical confounding situation. Here, we sampled an increasing number of features (1 to 100) with different χ^2 distribution given a binary confound (df = 3 (4) and scale= 0.5 (1) for confound= 0 (1)). The target was sampled from a normal distribution (M = 0, SD = 0.2) and the confound was added to it. Analysis of this data shows an increased performance after confound removal from M = −0.52 (SD = 0.02) to M = −0.50 (SD = 0.03) using one feature and from M = −0.02 (SD = 0.01) to M = 0.18 (SD = 0.01) using 100 features. These results demonstrate that the effect of confound-leakage increases with increasing number of features. These simulations show that skewed features, and by extension potentially other non-normal distributed features, can lead to confound-leakage. Interestingly, another consequence of non-normal distributions is insufficient removal of confounding information [21].

Confound-leakage due to limited precision features

A similar effect was observed with binary features, where unbalanced feature distributions conditioned on the confound led to leakage. Using simulations first we confirmed that a binary feature perfectly balanced in respect to the TaCo did not lead to confound-leakage (AUCROC of M = 0.50, SD = 0). Then, we repeated similar simulations but now we swapped two randomly selected distinct values of the feature within each CV-fold, preserving the marginal distribution of the feature but slightly changing its distribution conditional on the confound. This can be seen as adding a small amount of noise to the feature. Still, such a simple manipulation led to drastic leakage after TaCo removal with perfect AUCROC (M = 1.00, SD = 0.00), compared to AUCROC without CR (M = 0.52, SD = 0).

To further demonstrate this effect, we analyzed a simple demonstrative classification task using DT and two binary features derived from the UCI "heart dataset" representing the resting electrocardiographic (Restecg) results. Without CR the DT had 117 nodes and achieved a moderate AUCROC (M = 0.74, SD = 0.06). In stark contrast, after TaCo removal, the DT was extremely simple with only five nodes and achieved near-perfect AUROC (M = 0.99, SD = 0.01) (Fig. 3 E). Tellingly, this DT was able to make accurate predictions based on numerically minute differences in feature values. The reason for this becomes apparent when remembering that CR with a binary confound is equivalent to subtracting the mean of the corresponding confounding group from the respective feature. When applied to a binary feature, this results in four distinct values for a residual feature (Fig 3 E). When taken together with the results on the benchmark UCI data (see Analyses of benchmark data), we can see that such minute differences can be exploited by models such as DTs, RFs and MLPs but likely not by linear models. It is important to note, that leakage through minute differences was not only observed for binary features, but also other features with a limited precision (values containing only integer or with limited fractional parts). To demonstrate this, we predicted a random continuous target using either a normally distributed feature or the same feature rounded to the first digit. The original non-rounded feature performed at chance level both before (R^2 : M = −1.10, SD = 0.06) and after TaCo removal (R^2 : M = −1.03, SD = 0.07), while after rounding it lead to an improvement from M = −0.08 (SD = 0.01) to M = 0.70 (SD = 0.16) after TaCo removal. Features with limited precision, i.e. with no or rounded fractional part, are common, for instance, age in years, questionnaires in psychology and social sciences, and transcriptomic data.

Confound-leakage poses danger in clinical applications

ADHD is a common psychiatric disorder that is currently diagnosed based on symptomatology but objective computerized diagnosis is desirable [29]. Ideally a predictive model for diagnosing ADHD should not be biased by co-morbid conditions, e.g. depression [30]. To this end, comorbidity can be treated as a confound. However, a confound-leakage affected model, albeit with appealing performance, could lead to misleading diagnosis and treatment. To high-

light the danger of confound-leakage on this clinically relevant task, we analyzed a dataset with speech-derived features with the task to distinguish individuals with ADHD from controls. Our version of the dataset is a balanced subsample of the dataset described by Polier et. al. [3].

The baseline RF model without CR provided mean AUROC (M = 0.71, SD = 0.02). We then removed four confounds commonly considered for this task, age, sex, education level, and depression score (Beck's depression inventory, BDI), via featurewise CR in a CV-consistent manner. This resulted in a much higher AUCROC (M = 0.86, SD = 0.02). This model would be very attractive for real-world application if its performance is true–i.e. not impacted by leakage. However, as we have shown with our analyses confound-leakage can lead to such performance improvement. If confound-leakage is indeed driving the performance then this model could misclassify individuals as having ADHD because of confounding effects, e.g. their sex or depression, leading to misdiagnosis and wrong therapeutic interventions. To disentangle the effect of each confound, we looked at the performance after CR for each confound separately. Performing CR with BDI led to a high AUCROC with original features after CR (M = 0.91, SD = 0.01), shuffled features (M = 0.84, SD = 0.01). This result revealed that BDI is driving the potential leakage, owing to its strong relation to the target (Point-biserial correlation, r = 0.61, p < 0.01). Furthermore, a permutation test also led to the same conclusion (see Methods and Supplementary Fig. S2) Training CR models only on healthy individuals can be helpful in clinical applications [4]. We investigated this variant of CR and again the AUCROC increased for original features after CR M = 0.83 (SD = 0.02) and an increase with shuffled features from M = 0.51 (SD = 0.05) to M = 0.79 (SD = 0.02), suggesting that confound leakage is also a concern for variants of CR. Lastly, we wanted to evaluate why we observe confound-leakage on this dataset. The limited precision of features cannot be the reason here as all features are continuous. Therefore, we hypothesized that the confound leaked due to some features deviating from normal distributions. To this end we first compared the feature importance between the RF after CR and using the original features. Here, we observed the RFs' 10 most important features were completely different (Fig. 4 c-d), indicating that the two RF models rely on different relationships in the data. Next we visualized the distributions of the two most important features of the RF after CR for both models. This visualization (Fig. 4 e-f) clearly shows that CR has shifted the distributions due to deviations from normal distributions leaking information in their joint distribution. Furthermore, we trained new DTs using only these two features before or after CR. This led to an increase of AUCROC from 0.61 to 0.70 after CR only using these features. These analyses clearly demonstrate that real-world applications could suffer from confound-leakage and users should exercise care when implementing and validating a CR-ML workflow.

Discussion

Here, we exposed a hitherto unexplained pitfall in CR-ML workflows that use featurewise linear confound removal–a method popular in epidemiological and clinical applications. Specifically, we have shown this method can counter-intuitively introduce confounding, which can be exploited by some non-linear ML algorithms. Thus in addition to the already known pitfalls of residual confounding [21], our results show that CR may actually introduce confounding-information. We provide evidence of confound-leakage using a range of systematic controlled experiments on real and simulated data comprising both classification and regression tasks. First, to establish confound-leakage as opposed to information-reveal (of possibly nonlinear information) as the reason behind increased performance after CR, we proposed the TaCo framework, i.e., using the target as "superconfound". This extreme case of confounding allowed us to establish the existence, the extent, and possible mechanisms of confound-leakage. Specifically, by comparing the without CR baseline performance with CR after feature shuffling ($\tilde{X}_{CR}$) this framework can identify confound-leakage as the cause of increased predictive performance. We then extended the same framework to the more realistic scenario of weaker confounds showing that also there confound-leakage can occur.

To identify risk factors of confound-leakage, we performed several analyses. First, we demonstrated a mechanism by which confound-leakage can occur: differences of the conditional distributions of features given the confound. In the case of continuous features, non-normal distributions (e.g., skewed distributions) and in the case of discrete features, frequency imbalances can cause leakage, although other mechanisms could exist. Additionally, we show that features of limited precision (e.g., age in years and counts) also showed susceptibility due to this mechanism. Lastly, our results showed that the risk of confound-leakage increases with the number of features, which is especially problematic in the era of "big data", where tens of thousands of features are a norm.

Still we would like to highlight that we do not claim to have found all possible ways confound-leakage can happen. For instance, it is possible that other modeling approaches, even linear ones, could be susceptible to confound-leakage although we did not find evidence for it in our analyses. Nonetheless, confound-leakage can bias the data and may negatively impact subsequent statistical analysis [21].

It is important to note that although similar, confound-leakage is not equal to collider-bias. Colliders are variables causally influenced by both the features and target [19]. Both collider-bias and confound-leakage describe situations where variable adjustment can lead to spurious relationships between features and target. However, the collider bias assumes that the removed variable has to be caused by both the features and the target which is not shared by confound-leakage. One cannot exclude the possibility of collider removal using CR for many of our experiments as our operational definition of confounds does not include any assumption of causality. Still, we observe confound-leakage through CR for at least one causally defined confound (see walk-through analysis) and variables showing relationship only with the target. Such associations are not covered by the causal relationships described by a collider. In other words, the mechanisms of confound-leakage can lead to leaked information due to any variable related to the target and not only colliders or causal confounds.

Taken together, our extensive results show that the commonly used data types and settings of non-linear ML pipelines are susceptible to confound-leakage when using featurewise linear CR. Therefore, this method should be applied with care, and the ensuing models should be closely inspected, especially in critical decision domains. We concretely demonstrated this using an application scenario from precision medicine by building models for diagnosis of ADHD. We found that the attempt to control for comorbidity with depression using CR lead to confound-leakage. As many disorders often exhibit severe comorbidity, e.g., AHDH and depression as we demonstrated here but also neurodegenerative disorders are strongly confounded by ageing-related factors [31] as well as comorbidity in mental disorders [32, 33], the issue of confound-leakage should be carefully assessed in all such applications. We recommend the following best practices when applying CR together with non-linear ML algorithms:

1) Assess confounding strength: Check the confounds' relation to each feature and the target. In general, confounds strongly related to the target pose a greater danger of leaking predictive information. Here, we used a straightforward approach of measuring the correlations between the confound and target/feature. Other methods can be employed, e.g., proposed by Spisak [27]. Furthermore, measuring how dependent the predictions of a model are on the confound by permutation testing [34, 35] or the approach proposed by Dinga et al. [21] can be helpful.To gain additional information,

the reader might be interested in methods to estimate the variance in the target explained by ML predictions that confounds cannot explain [21, 27].

2) Compare performance with and without CR: If the performance increases after CR, one should investigate the reason behind the increase.

3) Gain evidence against or in favor of the confound-leakage: The procedure of shuffling the features followed by CR as we defined in the TaCo framework can provide clues regarding confound-leakage. Our shuffling approach can be seen as a single iteration of permutation testing. As our experiments suggest this is sufficient to obtain an indication of confound-leakage. However, a permutation test based null distribution can quantify the variability and provide additional information. It is important to note, however, that while this can provide evidence for confound-leakage, we are not aware of a procedure to definitively exclude confound-leakage as an explanation.

4) Carefully choose alternatives: If confound-leakage seems probable then consider alternative confound adjustment methods. Stratification [20, 36] is commonly in conventional machine-learning or unlearning of confounding effects [37] which is common in deep learning and further general approaches that promote fairness [12, 38]. Note however, that these procedures may also entail pitfalls. Hence, we caution researchers to exercise care when applying any confound adjustment protocol and to carefully consider limitations of the modeling approach used.

Conclusions and Future Directions

Important societal questions involving health and economic policy can be informed by applying powerful nonlinear ML models to large datasets. To draw appropriate conclusions, confounds must be removed without introducing new issues that cloud the results. In the present study, we performed extensive numerical experiments to gather evidence for confound-leakage. Using feature shuffling and predictions due to confound predicted features as proposed here, investigators can get an initial indication of whether their pipeline and data are susceptible to confound-leakage. We highlighted the conditions most likely to lead to leakage. Although we made progress on understanding these issues, there is no full-proof method for detecting and eliminating leakage. We hope our results prompt others to push further, perhaps expanding on the standard definition we adopted for confounds by introducing causal analyses. We hope our and allied efforts inform both researchers and practitioners who incorporate ML models into their data analyses. As a starting point, we suggest following the guidelines we provide to mitigate against confound-leakage.

Methods

Data

We analyzed several ML benchmark datasets from diverse domains to draw generalizable conclusions. To ensure reproducibility, most datasets come from the openly accessible UCI repository [39]. We included five classification tasks and five regression tasks with different sample sizes and numbers of features. All classification problems were binary or were binarized, and class labels were balanced to exclude biases due to class imbalance [40].

We also used one clinical dataset, a balanced subsample of the ADHD speech dataset described by von Polier et al. [3] includes 126 individuals with 6016 speech-related features, the binary target describing ADHD status (ADHD or control) and contains four confounds: gender, education level, age and, depression score measured using the Beck's depression inventory (BDI). For more information on the datasets see Supplementary Table S1.

Confound removal

Confound removal was performed following the standard way of using linear regression models. Following the common practice, we applied CR to all the features. Specifically, for each feature, a linear regression model was fit with the feature as the dependent variable and the confounds as independent variables. The residuals of these models, i.e., original feature minus the fitted values were used as confound-free features ($X_{CR} = X - \hat{X}$). This procedure was performed in a CV-consistent fashion, i.e., the confound removal models were fitted on the training folds and applied to the training and test folds [20, 22].

Target as a Confound (TaCo)

The TaCo framework allows systematic analysis of confound removal effects. Confounding is a three-way relationship between features, confounds and the target. This means that a confound needs to share variance with both the feature and the target. Measuring or simulating such relationships can be hard especially if linear univariate relationships cannot be assumed. Furthermore, effects of confound removal should increase with the actual strength of the confound. The target itself explains all the shared variance and thus it is the strongest possible confound. Therefore, using the target as a confound, i.e. TaCo, measures the most possible extent of confounding. In addition, using the TaCo simplifies the analysis to a two-way relationship. Lastly, the TaCo approach is applicable to any dataset and can help to measure the strongest possible extent of confound-leakage even without knowing the confounds.

Machine Learning Pipeline

To study the effect of CR on both linear and nonlinear ML algorithms, we employed a variety of algorithms: linear/logistic regression (LR), linear kernel Support-vector machine (linear SVM), Radial Basis Function kernel Support-vector machine (RBF SVM), decision tree (DT), random forest (RF), and multilayer perceptron (MLP) with a single hidden layer (relu). Additionally, we used dummy models to evaluate chance-level performance.

In the preprocessing steps, we normalized the continuous features and continuous confounds to have a mean of zero and unit variance, again in a CV-consistent fashion. Any categorical features were one-hot encoded following standard practice.

Evaluation

We compared the performance of ML pipelines with and without CR. To this end, we computed the out-of-sample Area under the Curve for ROC (AUCROC) for classification and predictive R^2 from scikit-learn [41] for regression problems in a 10 times repeated 5-fold nested CV. We employed the Bayesian ROPE approach [42] to determine whether the results for a given dataset and algorithm with and without CR were meaningfully higher, lower or not meaningfully different.

The Bayesian ROPE for model comparison

In this study we used the Bayesian ROPE [42] approach to qualify differences between K-fold cross-validation results coming from two models. This approach uses the Bayesian framework to compute probabilities of the metric falling into a defined region of practical equivalence or of one ML pipeline scoring higher than the other. This is achieved by defining a region of equivalence (here we used 0.05). Consequently, the Bayesian ROPE approach allows us to make probabilistic statements regarding whether and if so which of the ML pipelines score higher. We summarize these differences using

the following symbols = (highest probability of pipelines scoring practically equivalent), < (highest probability of right pipeline scoring higher), > (highest probability of left pipeline scoring higher). Other possibilities such as the significance test correcting for the dependency structure in K-fold CV [43] or permutation testing by shuffling the target or features can be employed when suitable.

Feature shuffling approach

Shuffling the features while keeping the confounds and target intact destroys the feature-target and feature-confound relationships while preserving the confound-target relationship. Therefore, after feature shuffling any confound adjustment method cannot reveal the feature-target relationship, but it can still leak information. In other words, any performance above the chance level after CR on shuffled features is an indication of confound-leakage. Feature shuffling is also used in other approaches such as permutation testing (see section Bayesian ROPE) to test effectiveness of confound adjustment methods [21]. Permutation testing can be computationally expensive and, like other frequentist tests, it cannot accept the null hypothesis to establish equivalence. We, therefore, adopted a computationally feasible methodology. We shuffle the features, perform repeated nested cross-validation and then apply the Bayesian ROPE. For completeness, we show that both permutation testing and the Bayesian ROPE detect confound leakage in the clinical dataset. In some cases feature shuffling approaches might need further consideration, for instance shuffling features within confound categories to preserve their joint distribution (see walk-through analysis), and the possibility of suppression and leakage happening simultaneously. Nevertheless, they serve as a useful tool for detecting confound leakage as shown in this work.

Availability of source code and requirements

- Project name: Confound-leakage
- Project home page: 'https://github.com/juaml/ConfoundLeakage'
- Operating system(s): GNU/Linux
- Programming language Python 3.10.8 [43]
- Other requirements: scikit-learn 0.24.2, baycomp 1.0.2, matplotlib 3.5.1, seaborn 0.11.2, dtreeviz 1.3.5, numpy 1.22.3, pandas 1.2.5
- License: GNU Affero General Public License v3.0

Availability of supporting data and materials

All 10 UCI benchmark datasets can be access freely at the UCI machine learning reporsitory [39]. Together with our simulated data (availabl under https://github.com/juaml/ConfoundLeakage) , the UCI benchmark datasets compose our minimal data sets to reproduce our key findings. Additionally, we analyzed one real-world clinical datase ([3]). This sensitive data is available from PeakProfiling GmbH with certain restrictions. Restrictions apply to the availability of the data, which were used under licence for this study. Please contact Jörg Langner the co-founder and CTO of PeakProfiling GmbH with requests.

Declarations

List of abbreviations

- ADHD: Attention Deficit Hyperactivity Disorder
- AUCROC: Receiver Operating Characteristic Curve
- BDI: Beck's Depression Inventory
- CR: Confound Regression
- CV: Cross-Validation
- DT: Decision Tree
- ML: Machine Learning
- MLP: Multilayer perceptron
- RBF: Radial Basis Function
- RF: Random Forest
- SVM: Support Vector Machine
- TaCo: Target as a Confound

Ethical Approval

All procedures contributing to this work comply with the ethical standards of the relevant national and institutional committees on human experimentation and with the Helsinki Declaration of 1975, as revised in 2008. The ADHD data collection and use involving human subjects/patients were approved by the ethics committee of the Charite Universitatsmedizin Berlin, Berlin, Germany, the approval number is EA4/014/10. All necessary patient/participant consent has been obtained and the appropriate institutional forms have been archived by the data collectors. The ethics protocols for analyses of these data were approved by the Heinrich Heine University Düsseldorf ethics committee (No. 4039, 4096).

Competing Interests

The Authors declare no Competing Financial or Non-Financial Interests but the following Personal Financial Interest: Georg G. von Polier participated and received payments in the national advisory board ADHD of Takeda.

Funding

This work was partly supported by the Helmholtz-AI project DeGen (ZT-I-PF-5-078), the Helmholtz Portfolio Theme 'Supercomputing and Modeling for the Human Brain' and Deutsche Forschungsgemeinschaft (DFG, German Research Foundation), project-ID 431549029 – SFB 1451 project B05.

Author's Contributions

Study concept and design: S.H., B.C.L, G.G.P., S.W., H.S., S.B.E., and K.R.P. Data collection and processing: G.G.P. for the ADHD data. Data analysis and interpretation: all authors. Drafting of the manuscript: S.H. Critical revision of the manuscript for important intellectual content and final approval: all authors. Supervision: B.C.L., S.B.E., and K.R.P.

Acknowledgements

We thank the UCI machine learning repository [39] and the original dataset contributors.

References

1. Zeng LL, Wang H, Hu P, Yang B, Pu W, Shen H, et al. Multi-Site Diagnostic Classification of Schizophrenia Using Discriminant Deep Learning with Functional Connectivity MRI. EBioMedicine 2018;30:74–85. https://www.sciencedirect.com/science/article/pii/S2352396418301014.
2. Qin K, Lei D, Pinaya WHL, Pan N, Li W, Zhu Z, et al. Using graph convolutional network to characterize individuals with major depressive disorder across multiple imaging sites. eBioMedicine 2022;78:103977. https://www.sciencedirect.com/science/article/pii/S235239642200161X.
3. von Polier GG, Ahlers E, Amunts J, Langner J, Patil KR, Eickhoff

SB, et al. Predicting adult Attention Deficit Hyperactivity Disorder (ADHD) using vocal acoustic features. medRxiv 2021 3;http://medrxiv.org/lookup/doi/10.1101/2021.03.18.21253108.
4. Dukart J, Schroeter ML, Mueller K. Age correction in Dementia - Matching to a healthy brain. PLoS ONE 2011;6.
5. Jo ES, Gebru T. Lessons from archives: Strategies for collecting sociocultural data in machine learning. In: Proceedings of the 2020 Conference on Fairness, Accountability, and Transparency ACM; 2020. p. 306–316. https://dl.acm.org/doi/10.1145/3351095.3372829.
6. Johnson WE, Li C, Rabinovic A. Adjusting batch effects in microarray expression data using empirical Bayes methods. Biostatistics 2007 1;8:118–127. http://dx.doi.org/10.1093/biostatistics/kxj037.
7. Whalen S, Schreiber J, Noble WS, Pollard KS. Navigating the pitfalls of applying machine learning in genomics. Nature Reviews Genetics 2022 3;23:169–181. https://www.nature.com/articles/s41576-021-00434-9.
8. Pomponio R, Erus G, Habes M, Doshi J, Srinivasan D, Mamourian E, et al. Harmonization of large MRI datasets for the analysis of brain imaging patterns throughout the lifespan. Neuroimage 2020 3;208:116450. http://dx.doi.org/10.1016/j.neuroimage.2019.116450.
9. Badgeley MA, Zech JR, Oakden-Rayner L, Glicksberg BS, Liu M, Gale W, et al. Deep learning predicts hip fracture using confounding patient and healthcare variables. npj Digital Medicine 2019;2.
10. Luders E, Toga AW, Thompson PM. Why size matters: differences in brain volume account for apparent sex differences in callosal anatomy: the sexual dimorphism of the corpus callosum. Neuroimage 2014 1;84:820–824. http://dx.doi.org/10.1016/j.neuroimage.2013.09.040.
11. Wiersch L, Hamdan S, Hoffstaedter F, Votinov M, Habel U, Clemens B, et al. Accurate sex prediction of cisgender and transgender individuals without brain size bias. bioRxiv 2022 1;p. 2022.07.26.499576. http://biorxiv.org/content/early/2022/07/28/2022.07.26.499576.abstract.
12. Mehrabi N, Morstatter F, Saxena N, Lerman K, Galstyan A. A Survey on Bias and Fairness in Machine Learning. ACM Computing Surveys 2021;54.
13. MacKinnon DP, Krull JL, Lockwood CM. Equivalence of the mediation, confounding and suppression effect. Prevention Science 2000;1.
14. Pourhoseingholi MA, Baghestani AR, Vahedi M. How to control confounding effects by statistical analysis. Gastroenterology and Hepatology from Bed to Bench 2012;5. https://www.ncbi.nlm.nih.gov/pubmed/24834204.
15. Deng J, Dong W, Socher R, Li LJ, Li K, Fei-Fei L. ImageNet: A large-scale hierarchical image database. In: 2009 IEEE Conference on Computer Vision and Pattern Recognition; 2009. p. 248–255.
16. Alfaro-Almagro F, McCarthy P, Afyouni S, Andersson JLR, Bastiani M, Miller KL, et al. Confound modelling in UK Biobank brain imaging. NeuroImage 2021;224.
17. Rao A, Monteiro JM, Mourao-Miranda J. Predictive modelling using neuroimaging data in the presence of confounds. NeuroImage 2017;150.
18. Chyzhyk D, Varoquaux G, Milham M, Thirion B. How to remove or control confounds in predictive models, with applications to brain biomarkers. GigaScience 2022;11.
19. Greenland S. Quantifying biases in causal models: Classical confounding vs collider-stratification bias. Epidemiology 2003;14.
20. Snoek L, Miletić S, Scholte HS. How to control for confounds in decoding analyses of neuroimaging data. NeuroImage 2019;184.
21. Dinga R, Schmaal L, Penninx BWJH, Veltman DJ, Marquand AF. Controlling for effects of confounding variables on machine learning predictions. bioRxiv 2020;.
22. More S, Eickhoff SB, Caspers J, Patil KR. Confound Removal and Normalization in Practice: A Neuroimaging Based Sex Prediction Case Study. In: Lecture Notes in Computer Science (including subseries Lecture Notes in Artificial Intelligence and Lecture Notes in Bioinformatics), vol. 12461 LNAI; 2021. p. 3–18.
23. Weele TJV, Shpitser I. On the definition of a confounder. Annals of Statistics 2013;41.
24. Dagaev N, Roads BD, Luo X, Barry DN, Patil KR, Love BC. A Too-Good-to-be-True Prior to Reduce Shortcut Reliance. Pattern Recognition Letters 2022;In press. https://arxiv.org/abs/2102.06406.
25. Geirhos R, Jacobsen JH, Michaelis C, Zemel R, Brendel W, Bethge M, et al. Shortcut learning in deep neural networks. Nature Machine Intelligence 2020 11;2:665–673. http://www.nature.com/articles/s42256-020-00257-z.
26. Nygaard V, Rødland EA, Hovig E. Methods that remove batch effects while retaining group differences may lead to exaggerated confidence in downstream analyses. Biostatistics 2016;17.
27. Spisak T. Statistical quantification of confounding bias in predictive modelling. CoRR 2021 11;abs/2111.00814. http://arxiv-export-lb.library.cornell.edu/abs/2111.00814.
28. Grinsztajn L, Oyallon E, Varoquaux G. Why do tree-based models still outperform deep learning on tabular data? arXiv 2022 7;https://arxiv.org/abs/2207.08815.
29. Gualtieri CT, Johnson LG. ADHD: Is Objective Diagnosis Possible? Psychiatry (Edgmont (Pa : Township)) 2005;2.
30. Katzman MA, Bilkey TS, Chokka PR, Fallu A, Klassen LJ. Adult ADHD and comorbid disorders: clinical implications of a dimensional approach. BMC Psychiatry 2017 8;17:302. http://dx.doi.org/10.1186/s12888-017-1463-3.
31. Wyss-Coray T. Ageing, neurodegeneration and brain rejuvenation. Nature 2016 11;539:180–186. http://dx.doi.org/10.1038/nature20411.
32. Joshi G, Wozniak J, Petty C, Martelon MK, Fried R, Bolfek A, et al. Psychiatric comorbidity and functioning in a clinically referred population of adults with autism spectrum disorders: a comparative study. Journal of Autism and Developmental Disorders 2013 6;43:1314–1325. http://dx.doi.org/10.1007/s10803-012-1679-5.
33. Plana-Ripoll O, Pedersen CB, Holtz Y, Benros ME, Dalsgaard S, de Jonge P, et al. Exploring comorbidity within mental disorders among a danish national population. JAMA psychiatry 2019 3;76:259–270. http://archpsyc.jamanetwork.com/article.aspx?doi=10.1001/jamapsychiatry.2018.3658.
34. Epstein MP, Duncan R, Jiang Y, Conneely KN, Allen AS, Satten GA. A permutation procedure to correct for confounders in case-control studies, including tests of rare variation. American Journal of Human Genetics 2012 8;91:215–223. http://dx.doi.org/10.1016/j.ajhg.2012.06.004.
35. Neto EC, Pratap A, Perumal TM, Tummalacherla M, Bot BM, Mangravite L, et al. Using permutations to assess confounding in machine learning applications for digital health. arXiv 2018;https://arxiv.org/abs/1811.11920.
36. McNamee R. Regression modelling and other methods to control confounding. Occupational and Environmental Medicine 2005;62.
37. Dinsdale NK, Jenkinson M, Namburete AIL. Deep learning-based unlearning of dataset bias for MRI harmonisation and confound removal. NeuroImage 2021;228.
38. Zhao Q, Adeli E, Pohl KM. Training confounder-free deep learning models for medical applications. Nature Communications 2020;11. http://dx.doi.org/10.1038/s41467-020-19784-9.
39. Dua D, Graff C, UCI Machine Learning Repository; 2017. http://archive.ics.uci.edu/ml.
40. Collell G, Prelec D, Patil KR. A simple plug-in bagging ensemble based on threshold-moving for classifying binary and multiclass imbalanced data. Neurocomputing 2018;275.
41. Buitinck L, Louppe G, Blondel M, Pedregosa F, Mueller A, Grisel O, et al. API design for machine learning software: experiences

from the scikit-learn project. In: ECML PKDD Workshop: Languages for Data Mining and Machine Learning; 2013. p. 108–122.
42. Benavoli A, Corani G, Demšar J, Zaffalon M. Time for a change: A tutorial for comparing multiple classifiers through Bayesian analysis. Journal of Machine Learning Research 2017;18.
43. Van Rossum G, Drake Jr FL. Python tutorial. Centrum voor Wiskunde en Informatica Amsterdam, The Netherlands; 1995.

Figure 1. A walk-through analysis demonstrating our analysis pipeline and confound-leakage using DT. The results shown here are on the 30% test split. For the binary classification walk-through using the bank investment dataset, a subset of the features used are shown before CR (a) and after CR (b). Induced DTs and their performance before (c) or after CR (d). The DT after CR (d) is based on minute differences in only two features and still performs nearly perfectly and better compared to the DT on raw data (c). The regression analysis walk-through using simulated data is depicted as feature-target relationships with the dotted line showing the predicted values (e,f). The non-normal distribution of the feature conditioned on the confound leaks information usable by the DT. Here, CR removes the linear relationship, as intended, but introduces a stronger non-linear one by shifting the distribution of X_{CR} given $confound = 0$ in-between the two peaks of X_{CR} given confound $= 1$ (f).

Figure 2. Performance on the UCI benchmark datasets when using raw vs CR features (a) and raw vs the predicted features given the confound/TaCo/$\hat{X}$ (b). The two columns correspond to: 1) TaCo removal with four ML algorithms (LR, DT, RF, MLP), and 2) CR with simulated confound with different correlation to the target (range 0.2-0.8) with RF. (a,b) show performance using the original features while (c,d) show the performance on shuffled features. To check whether a difference between the performance of two models is meaningful, we used the Bayesian ROPE approach to identify what is most probable: performance being higher before removal (<), being higher after removal (>) or equivalent (=) (see the Methods section for details). When using a linear model (LR) TaCo removal leads to reduction in prediction performance, as expected. In contrast, nonlinear models lead to a higher performance for all datasets. This increase could be either explained by confound removal revealing information already in the data (suppression) or confound removal leaking information into the features (confound-leakage). Shuffling the features destroys association between features and the target, therefore subsequent performance increase after TaCo removal indicates the possibility of confound-leakage (c,d). The simulated confounds show that an increase after CR is also possible for confounds weakly related to the target (b,d) and one dataset (Blood) shows strong evidence of confound-leakage.

Figure 3. Two mechanisms for confound-leakage. First mechanism where non-normal distributions get shifted apart through CR. (a,b) show this using a simulation with extreme values on opposing sides for one feature conditioned on the TaCo. (c,d) show a simplified version (binary target for visualization purposes) of the house price UCI benchmark dataset. Here, the distributions of the feature conditional on the TaCo are different (c); a narrow distribution (TaCo = 1) and a distribution with two peaks (TaCo = 0). TaCo removal shifts the narrow distribution in-between the two peaks (d), leaking information usable by non-linear ML algorithms. The second mechanism, leakage through minute differences in the feature after CR, is highlighted through the visualization of the DT trained on the heart dataset after CR (e). Distribution plots visualize the data at each decision node. The decision boundary is shown as a dotted line. For decision nodes before leaf nodes, the side of the decision node leading into a prediction is colored to represent the predicted label as diagnosed (green) or not (purple). The minute differences in the two used features that perfectly separate the data into the two classes can be seen.

Figure 4. The real-world ADHD speech dataset. The performance when using different confounds (a-b), most important features of RF when using BDI as confound (c-d) and visualization of confound-leakage due to deviation from normal distributions (e-f). a shows the performance of a RF predicting ADHD vs healthy controls using the original features. To check whether a difference is meaningful we used the Bayesian ROPE approach to identify what is most probable: performance being higher before removal (<), being higher after removal (>) or equivalent (=) (see method section). An increased performance can be observed when using all confounds, BDI as a confound or the TaCo. The same pattern appears when the features were shuffled (b). This shows that the increase in performance is due to confound-leakage and BDI is a driving factor for this leakage as it leaks information when used as a confound. c-d visualize the 10 most important features for both using X and X_{CR} as features. The feature ranking is shown as white label on top of each cell. The most important features are different for X and X_{CR}. Furthermore, the most important features of one model ranked as very unimportant in the other. e-f show decision boundaries of DT trained on the two most important features after CR. The background colors indicate the prediction of the model, the points show the true target value and the x-axis the two most important features. The distribution of each feature conditioned on the target is shown as the density plots. One can see that CR leaks information by cleanly separating the blue and red points.

Figure 5. Prediction performance of a RF trained with (blue) or without (red) confound removal on an increasing number of features. Each feature was either sampled from a random standard normal distribution ($M = 0$, std=1), a random χ^2 distribution with $df = 3$ or a χ^2 distribution with a $df = 3$, scale= 0.5 or $df = 4$, scale= 1 for the confound being equal to 0 and 1 respectively. a) The RF trained on the normally distributed features did not achieve performance above the chance level ($R^2 < 0$) irrespective of confound removal. b-c) When training the RF on either of the χ^2 distributed features, confound removal resulted in above chance level performance ($R^2 > 0$). This effect increased with an increasing number of features and can only be explained by confound removal leaking information into the features.

Figure Click here to access/download;Figure;1.pdf

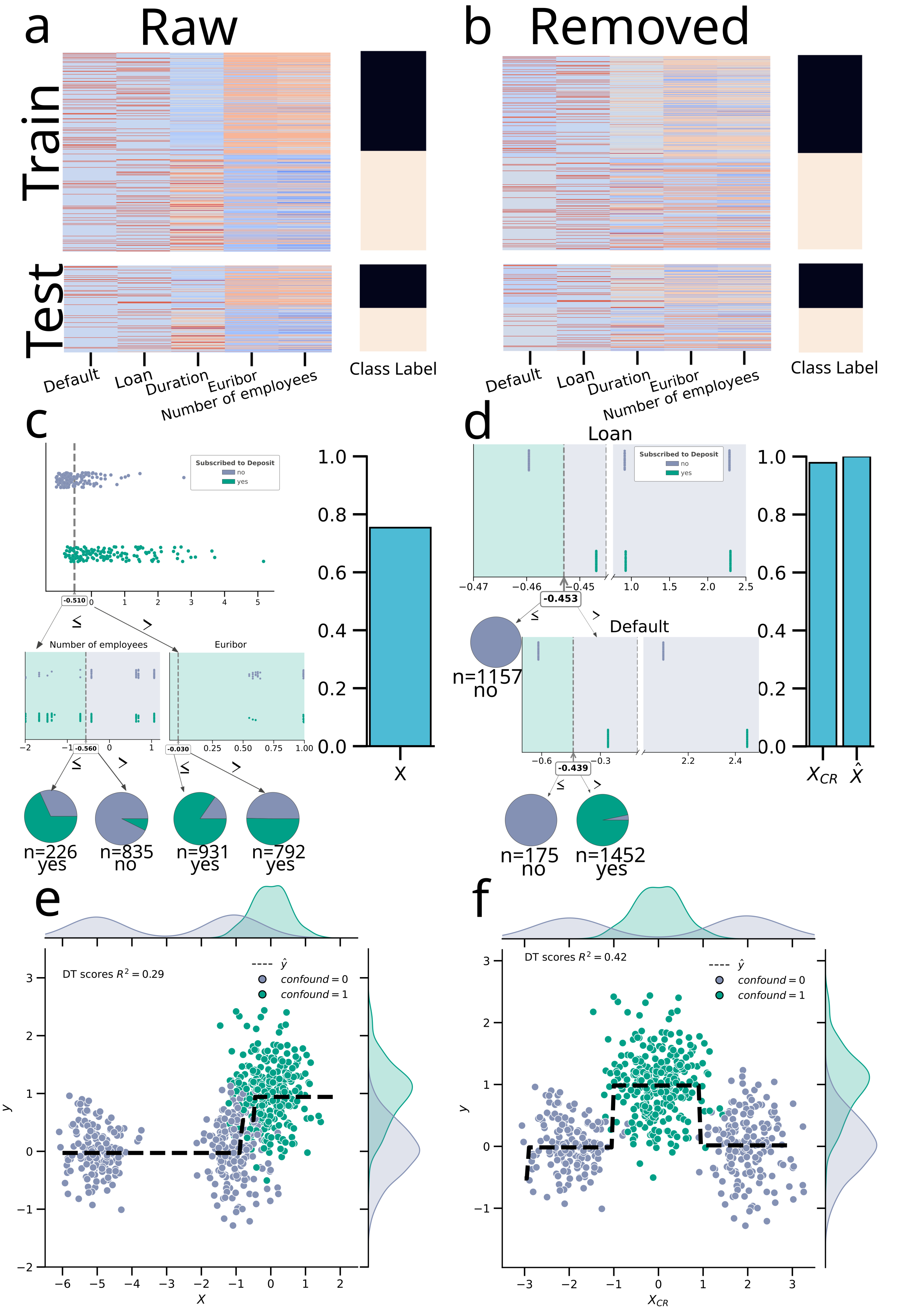

Click here to access/download;Figure;2.pdf

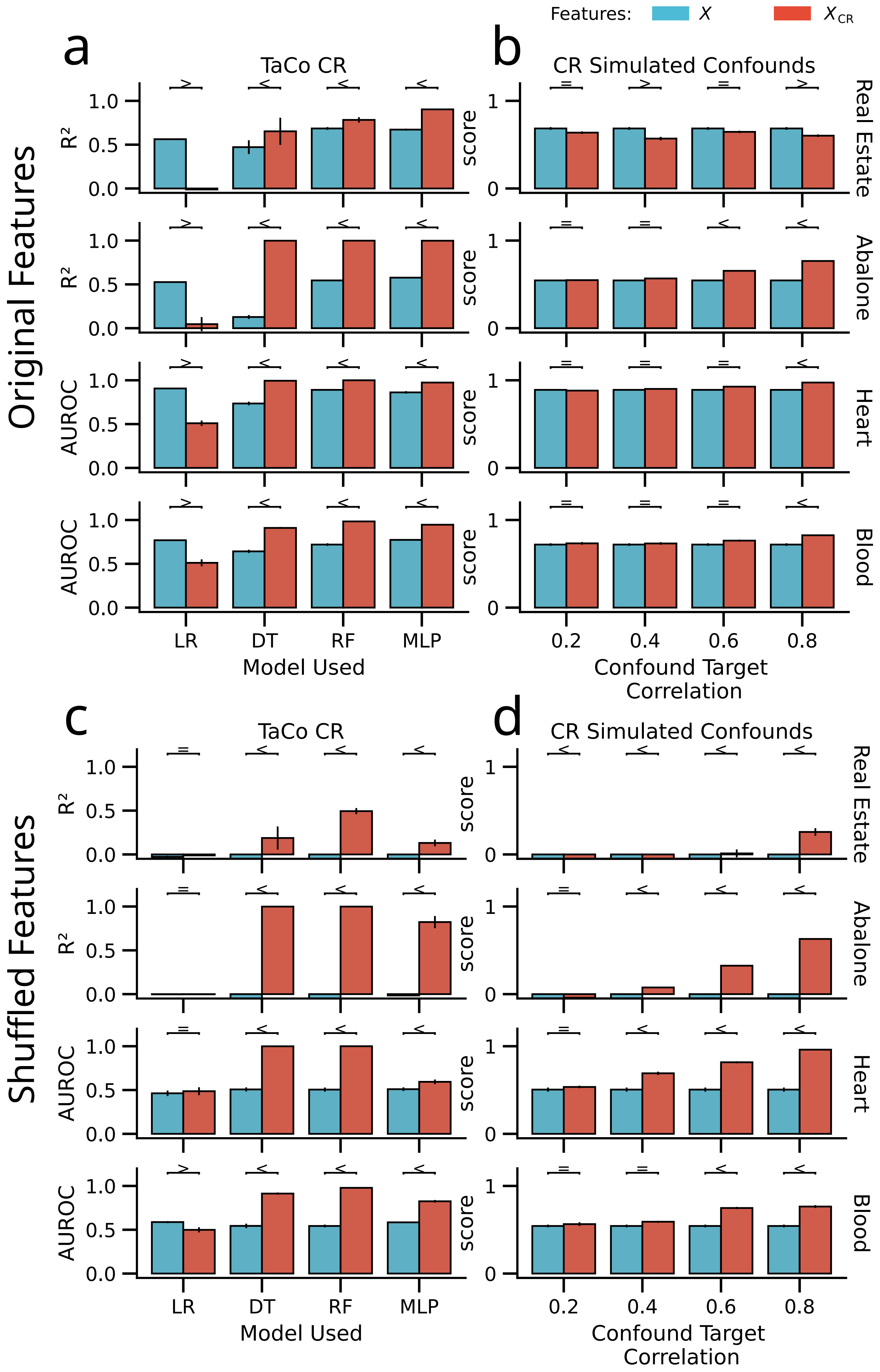

Click here to access/download;Figure;3.pdf

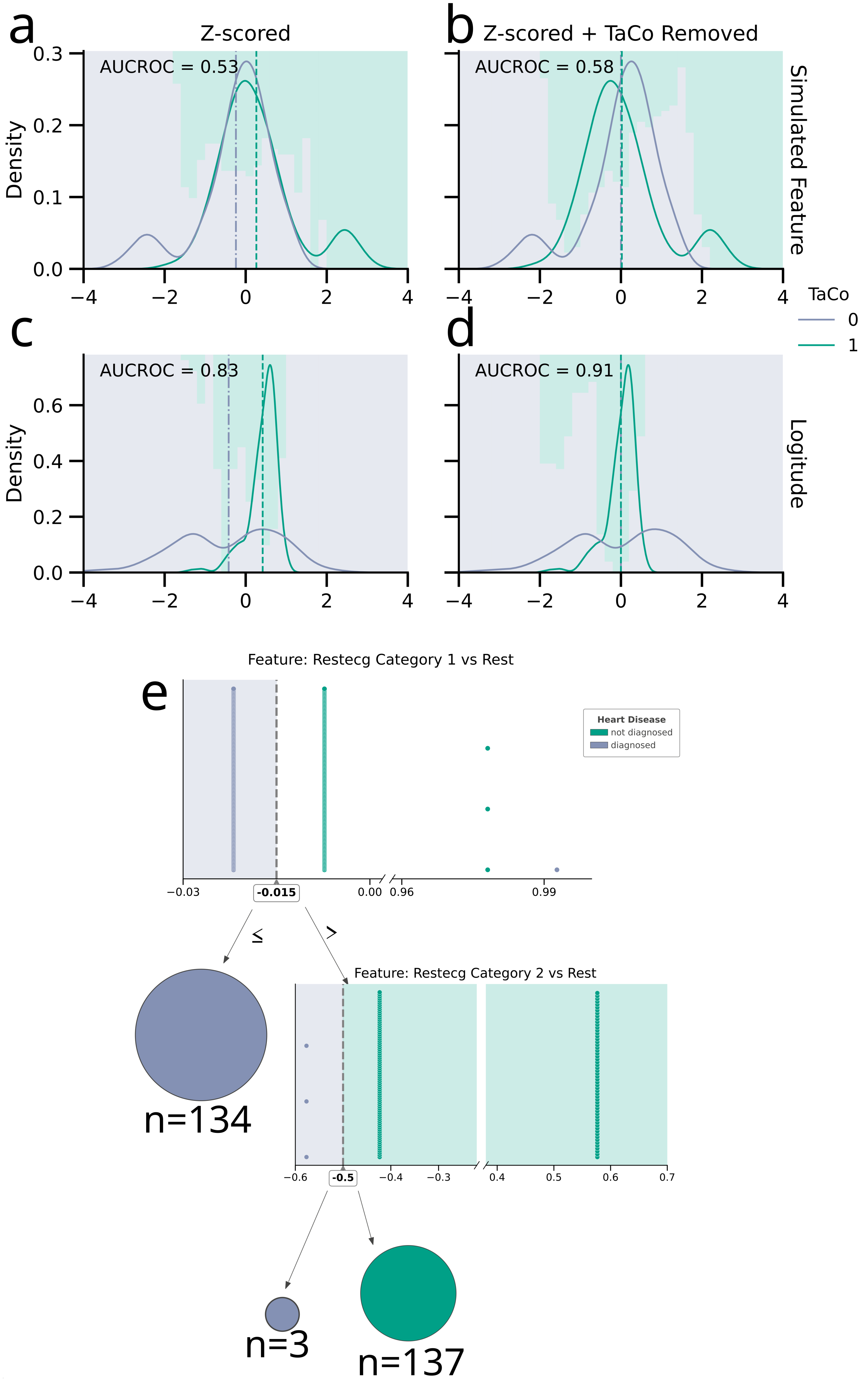

Figure

Click here to access/download;Figure;4.pdf

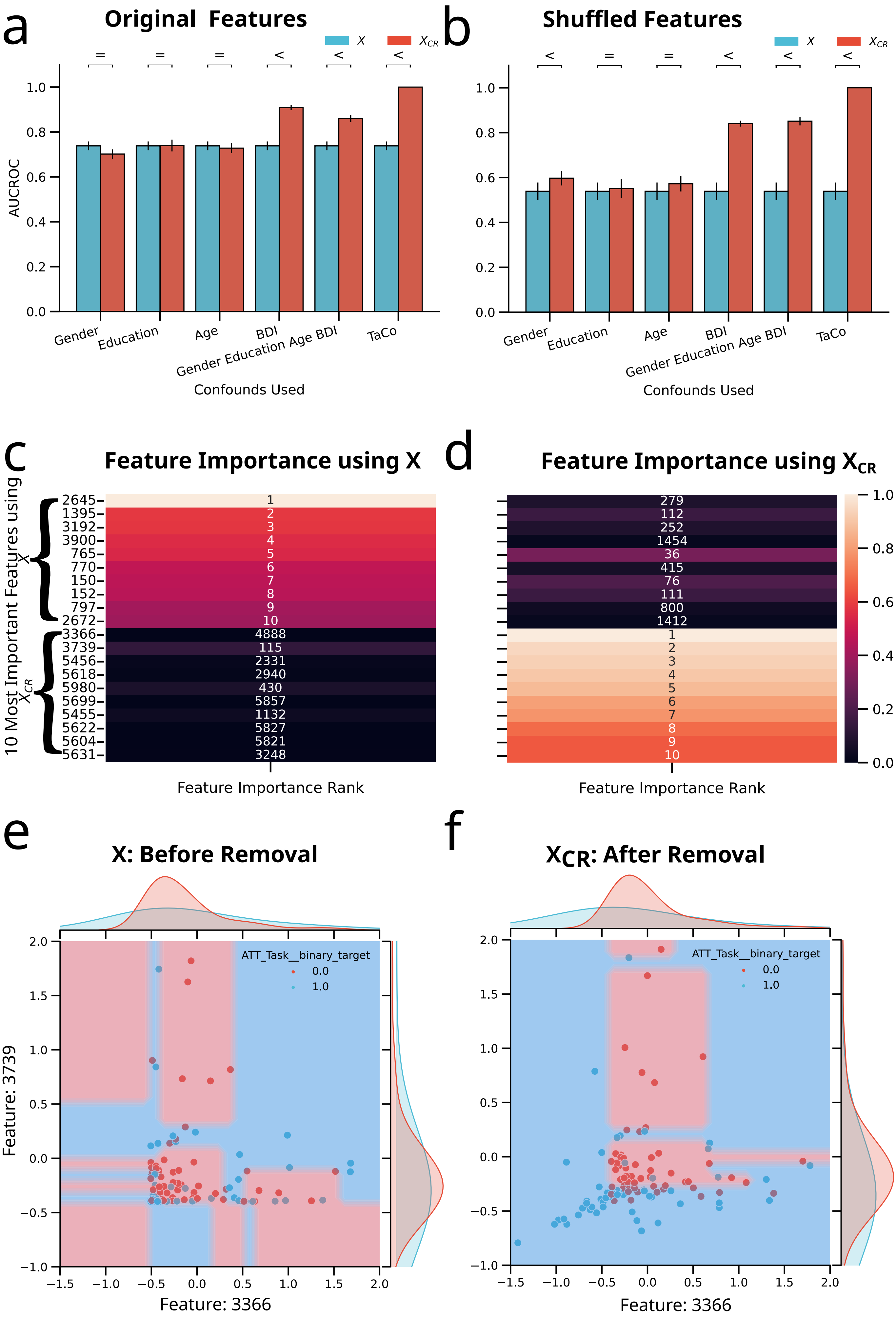

 Click here to access/download;Figure;5.pdf

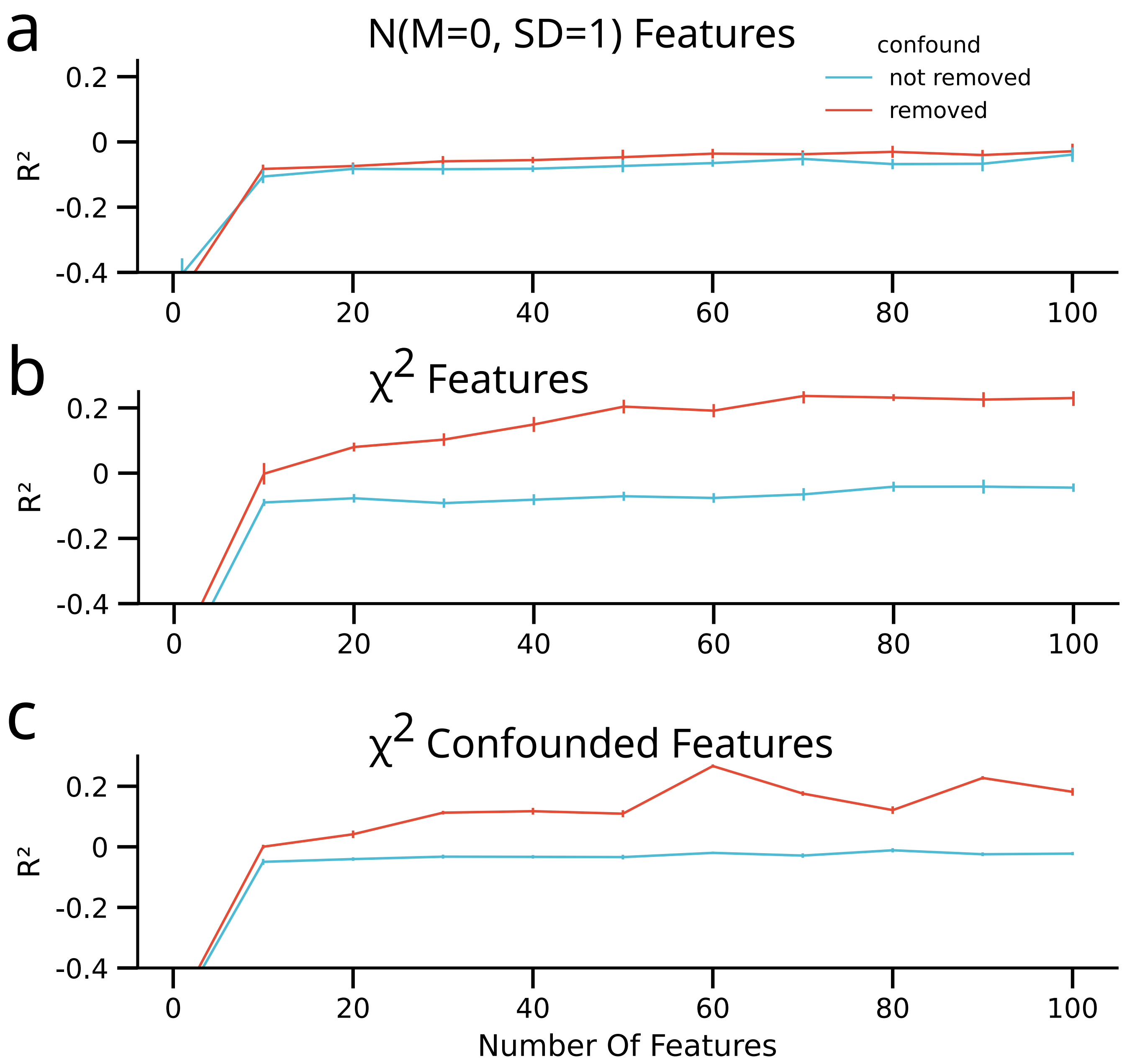

Click here to access/download
Supplementary Material
Pitfalls_of_Confound_Regression_Supplement_Revision
.pdf

Personal Cover
Click here to access/download;Personal Cover;Pitfalls_of_Confound_Regression_Cover_Letter_Revisio

31.05.2023

Re: Manuscript Revision for GigaScience GIGA-D-23-00004

Dear Dr. Scott Edmunds, dear Dr. Hans Zauner

I am writing to submit the revised version of our manuscript titled “Confound-leakage: Confound Removal in Machine Learning Leads to Leakage” for consideration for publication in GigaScience. We would like to express our gratitude for the valuable feedback provided by the reviewers, which has greatly contributed to improving the quality and rigor of our work.

In response to the reviewers' comments, we have made extensive revisions to the manuscript, incorporating additional analyses and simulations to strengthen our findings. We believe that these revisions have significantly enhanced the original manuscript and have addressed the concerns raised by the reviewers in a comprehensive manner, making it more valuable to the readers of GigaScience.

Specifically, we have conducted a series of additional experiments and simulations to further investigate the issue of "confound leakage". These analyses have allowed us to explore the impact of confound leakage and provide more robust evidence for the validity of our main findings. Importantly, while we have refined our methodology and provided additional evidence, the core message regarding the potential confound leakage remains consistent with our original submission. The code used for the additional analyses has also been deposited in the GitHub repository (https://github.com/juaml/ConfoundLeakage). We believe that our study contributes significantly to the existing literature and advances the understanding of data leakage in machine learning applications.

Below is a summary of the key changes we have made in response to the reviewers' comments:

1. Expanded Methodology: We have added two new sections in the manuscript to describe the analyses methods: “The Bayesian ROPE for model comparison” and “Target as a Confound (TaCo)”. These sub sections provide a detailed explanation of the methods employed, ensuring transparency and reproducibility.
2. Revised figures and a new figure: We have revised the figures that present the results of the additional analyses and simulations. These visual aids enhance the clarity and accessibility of our findings, allowing readers to better understand the impact of confound leakage. We have also added Figure 5, that shows the impact of an increasing number of features.
3. Discussion of implications: In the revised manuscript, we have further elaborated on the implications of the potential confound leakage. We have also discussed the limitations associated with this issue and provided suggestions for future research to minimize such effects.

Thank you once again for the opportunity to revise and resubmit our manuscript. We appreciate the time and effort invested by the reviewers in providing constructive comments, which have undoubtedly improved the quality of our work. We look forward to your favorable consideration of our manuscript for publication in GigaScience.

Should you require any additional information or have any further queries, please do not hesitate to contact me. Thank you for your attention, and we remain at your disposal.

Sincerely,

Sami Hamdan
PhD-student
Institute of Neuroscience and Medicine,
Brain & Behaviour (INM-7)
Research Centre Jülich, Germany
Email: s.hamdan@fz-juelich.de

Kaustubh R. Patil, PhD
Research Group Leader
Applied Machine Learning
Institute of Neuroscience and Medicine,
Brain & Behaviour (INM-7)
Research Centre Jülich, Germany
E-Mail: k.patil@fz-juelich.de

Personal Cover

Click here to access/download;Personal Cover;Pitfalls_of_Confound_Regression_Response_Letter_Re

Dear reviewers and GigaScience Editors,

Thank you very much for your feedback, review and the opportunity to revise our manuscript. In the following we first summarize our efforts to improve the manuscript and then provide a point-by-point response to all the comments. For this reason, we will always first display the reviewers' comment in blue color and our response in black color. Quotes from the manuscript and any changes are shown in triple quotes ("““ “””). Additional text is shown in green color and deleted text is shown striked through in red color.

Summary:
We thank both reviewers for their insightful feedback. We have addressed all the comments and this has led to an improved manuscript. We performed several additional analyses and sanity checks such as permutation testing and training confound removal models only on controls. We have removed our claims about use of X_hat, added new visualizations and extended the methods section making our findings more accessible. Our main claim that confound leakage can happen and adversely impact machine learning outcomes did not change. In fact, the additional extensive analyses including permutation testing and an analysis of the most important features in the clinical dataset substantiate our claims. Furthermore, these analyses highlight the impact of confound-leakage on real world data. In sum, we are very grateful for the reviewers feedback and the editors initiative that helped us improve our work.

Reviewer 1:

We thank Dr. Richard Dinga for the encouraging words and for the detailed comments. We have performed several sanity checks and other analyses to address the comments raised. The code used for those has been made available in the project's GitHub repository: https://github.com/juaml/ConfoundLeakage/blob/main/extra/check_auc/CheckAUC.pdf.

Reviewer (R.) 1 Comment (C.) 1:
Confound leakage due to implementation of performance measures: One of the main pieces of evidence for the "confound leakage" in the paper is that after shuffling features that there should be no relationship between features and the outcome, but after applying confound regression (CR), ML models can predict the outcome with above-chance performance. In my opinion, the explanation authors provide makes sense, and the data do produce this behavior. However, This is a tricky problem, and I might be wrong here, but I think that this bias only happens because performance measures implemented in scikit-learn (and other libraries, I am sure as well) always report above-chance performance, so AUC will never be < 0.5 and R2 < 0 (i.e., if model predictions would result to AUC = 0.98 or 0.02, scikit-learn will in both cases report 0.98). The bias, as described in the paper, does happen, but half the time, it's positive and half the time negative, and in the long run, it will average to 0. (See also my next point).
To elaborate, after feature shuffling, there should be no relationship between the features and outcome or features and confounds, so there should be no way to learn something in the training set that would translate to above-chance predictions on the test set. Confound removal will create a confound-dependent bias and hence a spurious signal in training set that a model will learn. However, in the test set, this bias will randomly half the time be in the opposite direction. So in the test set, the confound removal induced bias will half the time lead to significantly spuriously above chance and half the time below chance prediction performance. This does not mean that using different AUC implementations would solve the problem or that the problem is less severe, as described in the

paper. Results in each test set will still be biased (positively or negatively), with possibly severe consequences for the validity of the results. This should be explored and explained in the paper, and the recommendations should be changed accordingly.

Response R.1 C.1:
We address this comment in two parts.

A. Performance measure implementation in scikit-learn is correct
The reviewer argues that scikit-learn implementation AUC and R2 measures always report above-chance performance, which seems to be something happening in different ML libraries. We were not aware of this pitfall in ML libraries and want to thank the reviewer for raising this concern. However, as we show this is not the case in the scikit-learn version that we are using. We used scikit-learn (version>=0.24.2) and worked with simulated data as nicely provided by the reviewer in the next comment (see below, R.1 C.2). We generated 100 simulated datasets each using a different random seed. Then we computed the AUC in both R and Python in the same way as done by the reviewer, i.e we calculated the AUC of the original feature X and y and confound removed feature X_CR and y. Again, following the reviewer's example this was done without fitting a predictive model.

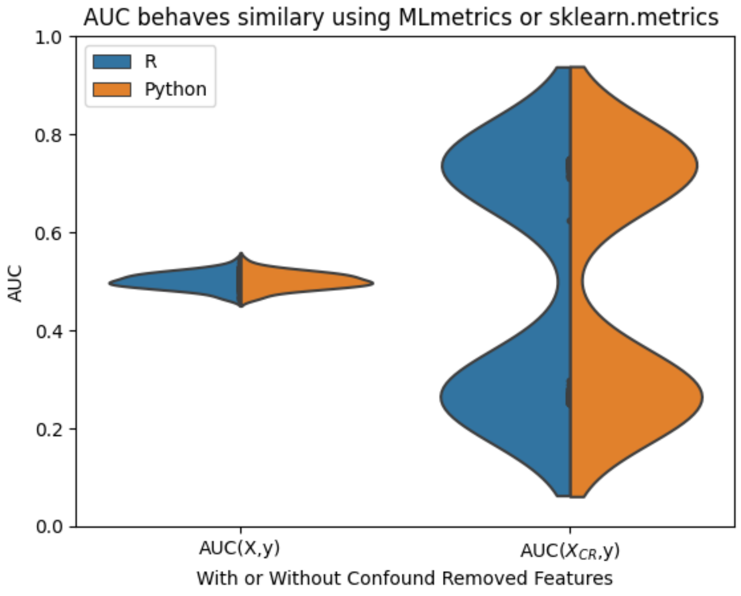

Both R and Python behave in the correct way as shown in the plot above. Specifically, they showed chance-level performance when using the original feature X (left hand side) but a bimodal distribution centered around 0.75 and 0.25 when using the confound removed feature X_CR. Note that both R and Python show below chance-level AUC. Thus our results, i.e. increased test performance after CR, can not be due to incorrect implementation of AUC in scikit-learn.
Along similar lines, we would like to note that scikit-learn's implementation of R2 (r2_score function) returns the coefficient of determination and not the squared correlation. As the documentation says (and we have confirmed it) "Best possible [R2] score is 1.0 and it can be negative (because the model can be arbitrarily worse)." Further details can be found in the scikit-learn documentation:
https://scikit-learn.org/stable/modules/generated/sklearn.metrics.r2_score.html.

or check directly in the source code starting here:
https://github.com/scikit-learn/scikit-learn/blob/364c77e047ca08a95862becf40a04fe9d4cd2c98/sklearn/metrics/_regression.py#L927

B. Confound-dependent bias in the test set is not random
The second part of the comment raises the possibility that confound-dependent bias introduced after confound removal in the training set would lead to random behavior in the test set–i.e. sometimes lower and sometimes higher than chance-level performance. The concern here is that if a particular implementation always returns above chance performance then such randomness would be seen as high performance instead of averaging out to chance level.
As we have shown above the scikit-learn implementation we used does indeed return both below and above chance-level performance and hence we believe that this concern is not applicable. The concern regarding behavior on the test set is addressed in the next comment for brevity reasons.

Reviewer (R.) 1 Comment (C.) 2:
Confound regression in linear models: I think the problems of CR, as described in this paper, will also be present in linear models, thus making the findings more impactful, and it should be added to the paper. Here is an example with categorical data, if you excuse R code:
```rwe
set.seed(1)
n <- 1000
df <- data.frame("x" = rep(c(0,1), n),
"y" = rbinom(n, 1, 0.25))
df$x_cr <- resid(lm(x~y, data=df))
library(MLmetrics)
MLmetrics::AUC(df$x_cr, df$y)
MLmetrics::AUC(df$x, df$y)
```
Here I do not fit any model, and I do not bother with the test set, but the AUC of a linear model fitted on x or x_cr (feature x, after the confound removal) will be the same as the AUC of x or x_cr variables themself. Also, notice that with different seeds, AUC will sometimes be 0.25 and sometimes 0.75, although scikit-learn would report 0.75, following the previous point.
Basically, the confound regression applied to a binary variable will shift the variable slightly with respect to the confound/target, thus increasing the number of unique values of the variable and creating a correlation between the variable and the outcome. Since the shift is random, the correlation will also be random, sometimes positive and sometimes negative. Also, in [1], we describe situations where linear models can learn confounding information from the data after confound cleaning, especially robust linear models, and I suspect similar issues can also lead to biases described in this manuscript.

Response R.1 C.2:
Thank you very much for this detailed comment and for providing a R code snippet which helped us understand your concern.
It is unclear to us why the AUC given a feature and target (as computed by the reviewer) should be the same when using a prediction model. To investigate this, using the same 100 datasets generated above, we fitted a logistic regression model with either X or X_CR as a feature. As can be seen in the plot below, the AUC of predicted values (indicated as y_hat) is

close to the chance level for both X and X_CR (blue and orange leftmost boxplots). Then we fitted decision tree models to the same data and while the AUC on the original feature stayed at the chance level, that of the confound removed feature was much higher (rightmost boxplot, mean close to 1).

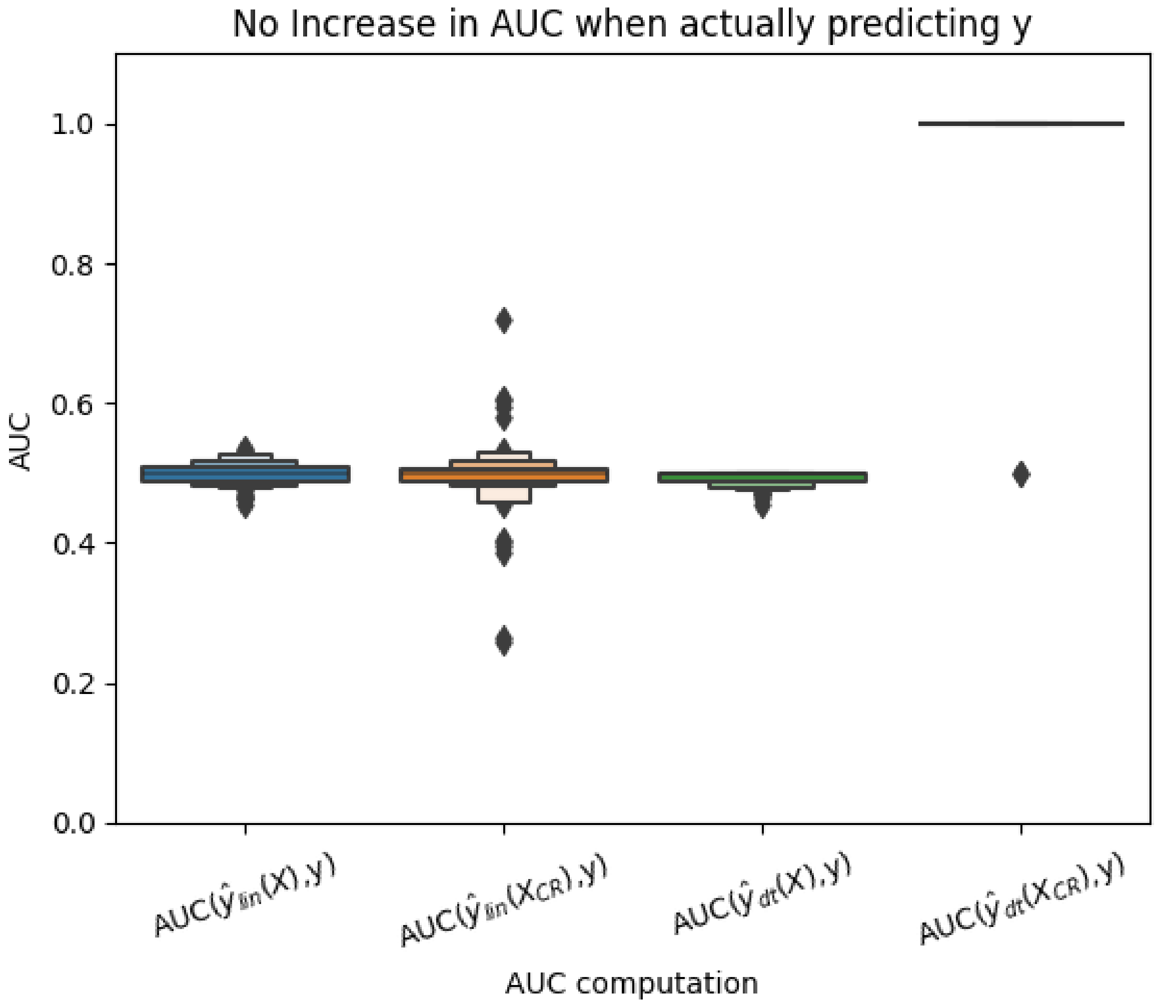

Here, you can see the AUC of either X and X_CR or predicted y (y_hat) given either the raw (X) or confound removed (X_CR) features. As you can see the previously described effect is not anymore visible when using the predicted y for AUC computation. Overall, the linear model (logistic regression) did not learn the confound-dependent bias and performed at the chance level (orange plot above) while a nonlinear model (decision tree) managed to learn the confound-dependent signal resulting in high AUC (rightmost boxplot above). Also note that the performance after model fitting does not fluctuate between below and above chance level values as it does when using the original feature (see the response to R.1 C.1 above). Furthermore, we would like to note that [1] discusses confounding left in the data after confound removal while we investigate introduction of confounding effects through CR. The analysis performed here uses all the data, i.e. no separate train and test sets, to align with the reviewer's line of thought but in the manuscript we exclusively perform predictions on hold-out data.
We agree with the reviewer that an effect if found in linear models would increase the impact of the finding but given the correct behavior of scikit-learn and chance-level performance when using a linear prediction model we find no evidence for this. However, we cannot rule

out that in some cases linear models might learn confound-dependent biases. To highlight this we have added the following sentences to the manuscript (section: Discussion page 5):
“”””
which is especially problematic in the era of “big data”, where tens of thousands of features are a norm.
Still we would like to highlight that we do not claim to have found all possible ways confound-leakage can happen. For instance, it is possible that other modeling approaches, even linear ones, could be susceptible to confound-leakage although we did not find evidence for it in our analyses. Nonetheless, confound-leakage can bias the data and may negatively impact subsequent statistical analysis [21].
It is important to note that although similar, confound-leakage is not equal to collider-bias. Colliders are variables causally influenced by both the features and target [19].
“”””

R. 1 C. 3:
Permutation test: The biases caused by confound regression (positive and negative, or just positive) should also be presented in the permutation distribution, so using a permutation test will not change the bias of the results, but the results will at least not be statistically significant, so it can guard in some way against this problem. Also, an inspection of the permutation distribution might warn researchers about possible problems as described in the paper, e.g., if the distribution is not centered at 0 or is bimodal, it might suggest the "confound leakage." Although the permutation test is not always computationally feasible, I think it should be discussed in the paper. Another way how a permutation test can help is by using stratified shuffling, where shuffling is performed only within confound categories.

Response R. 1 C. 3:
Thank you very much for this important remark.

Our goal in this paper was to investigate confound leakage where predictions using features after CR are using confounding information instead of actual signal within the features. Thus if the features are shuffled before CR there should be no information available to learn in the features apart from any leakage during CR. Following this logic, we show that shuffled features together with Bayesian ROPE can indeed detect confound leakage in various settings. As you note, permutation testing can also be used to detect confound leakage, with just positive bias as discussed above, and potentially provide additional information.

To address your suggestion we have taken the following actions:

A. We describe the Bayesian ROPE approach and the rationale behind using it in more detail.
B. We performed a permutation test on the clinically relevant dataset which also shows confound leakage.
C. We have added more details on permutation testing together with stratified shuffling.

We use the Bayesian ROPE approach which is a computationally more efficient method. Furthermore, it allows statements of equivalence which typically cannot be obtained in frequentist approaches. To make this clearer we added the following subsection to Methods about why we chose the Bayesian ROPE approach:

“”””

The Bayesian ROPE for model comparison

In this study we used the Bayesian ROPE [42] approach to qualify differences between K-fold cross-validation results coming from two models. This approach uses the Bayesian framework to compute probabilities of the metric falling into a defined region of practical equivalence or of one ML pipeline scoring higher than the other. This is achieved by defining a region of equivalence (here we used 0.05). Consequently, the Bayesian ROPE approach allows us to make probabilistic statements regarding whether and if so which of the ML pipelines score higher. We summarize these differences using the following symbols = (highest probability of pipelines scoring practically equivalent), < (highest probability of right pipeline scoring higher), > (highest probability of left pipeline scoring higher). Other possibilities such as the significance test correcting for the dependency structure in K-fold CV [43] or permutation testing by shuffling the target or features can be employed when suitable.

“”””

We do agree with the reviewer that permutation testing can provide additional information but it comes at an additional computational cost. To clarify this we have added the following sentence to the Discussion (page 6):

“”””

3) Gain evidence against or in favor of the confound-leakage:
The procedure of shuffling the features followed by CR as we defined in the TaCo framework can provide clues regarding confound-leakage.
Our shuffling approach can be seen as a single iteration of permutation testing. As our experiments suggest this is sufficient to obtain an indication of confound-leakage. However, a permutation test based null distribution can quantify the variability and provide additional information.

“”””

We have also added the following to results section (Walk-through analysis, page 3) to further highlight this:

“”””

To destroy the feature-target relationship we shuffled each feature before CR ($\breve{X}$) to create $\breve{X}$CR and repeated the analysis. As there should be no predictive information in the shuffled features, the only explanation for above chance-level performance is CR leaking information into the confound-removed features X_CR, i.e. confound-leakage. We applied the shuffling procedure to a train-test split in this walk through analysis. But it should be noted that when combined with a (nested) cross-validation and Bayesian ROPE approach, this procedure can be used to compare models similarly as a permutation test (see section Shuffling the features and permutation testing). We observed chance-level performance without CR (AUCROC = 0.52) for the shuffled features.

“”””

We were also happy to perform a permutation test on the clinically relevant dataset. The result is shown below and has been added as the new Supplementary Fig. S2.

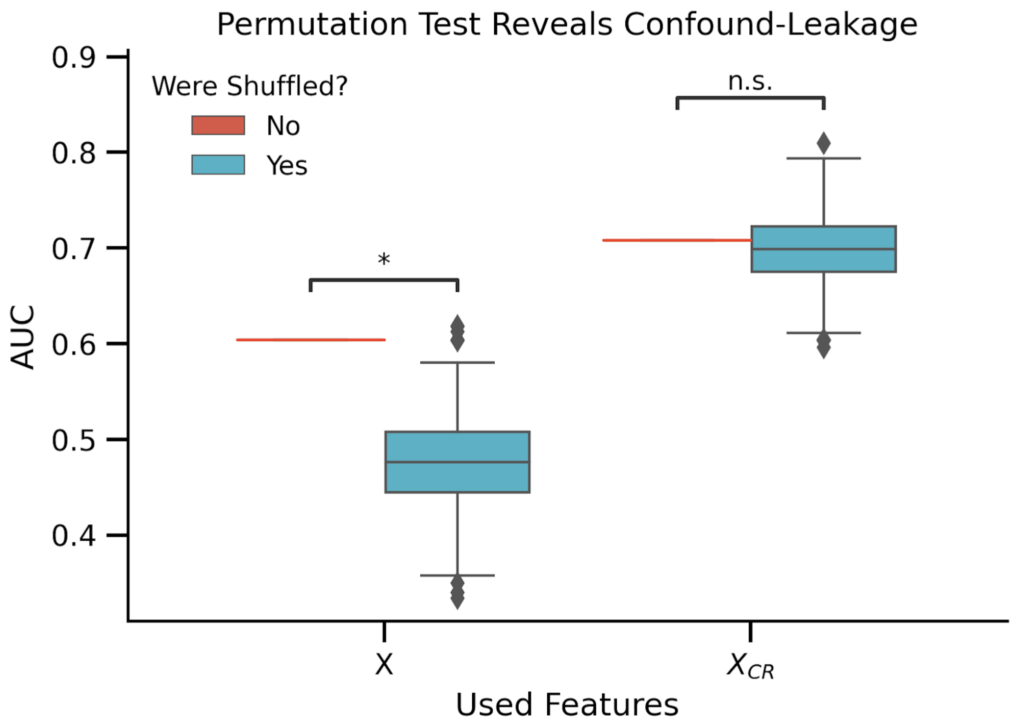

Figure S2: We performed permutation testing with 1000 iterations. After shuffling the features, a significantly lower performance was observed compared to the original features X. No significant difference between raw and shuffled features was observed when using the X_CR features. This result is in line with the leakage hypothesis as the higher accuracy after shuffling and CR indicates leaking target-related confounding information into the features.

‘’’

This result revealed that BDI is driving the potential leakage, owing to its strong relation to the target (Point-biserial correlation, $r = 0.61$, $p < 0.01$). Furthermore, a permutation test also led to the same conclusion (see Methods and Supplementary Fig. S2)

‘’’

Regarding stratified shuffling, again we agree that it can be a valuable approach, however, it could be difficult to implement with multiple confounds where it is hard to define clear categories (e.g. continuous variables). Furthermore, it is important to note that both approaches (Bayesian ROPE and permutation testing) should be used with care. Both will not be able to differentiate between leakage and the real signal present in the feature. Of course, in this case the performance of shuffled X_CR should still be higher than the chance level. For this reason we agree that inspecting the permutation distribution can be helpful. To address these and additional remarks, we have added the following subsection to the Methods section:

“”””

Feature shuffling approach

Shuffling the features while keeping the confounds and target intact destroys the feature-target and feature-confound relationships while preserving the confound-target

relationship. Therefore, after feature shuffling any confound adjustment method cannot reveal the feature-target relationship, but it can still leak information. In other words, any performance above the chance level after CR on shuffled features is an indication of confound-leakage. Feature shuffling is also used in other approaches such as permutation testing (see section Bayesian ROPE) to test effectiveness of confound adjustment methods [21]. Permutation testing can be computationally expensive and, like other frequentist tests, it cannot accept the null hypothesis to establish equivalence. We, therefore, adopted a computationally feasible methodology. We shuffle the features, perform repeated nested cross-validation and then apply the Bayesian ROPE. For completeness, we show that both permutation testing and the Bayesian ROPE detect confound leakage in the clinical dataset. In some cases feature shuffling approaches might need further consideration, for instance shuffling features within confound categories to preserve their joint distribution (see walk-through analysis), and the possibility of suppression and leakage happening simultaneously. Nevertheless, they serve as a useful tool for detecting confound leakage as shown in this work.

“””

R. 1 C. 4:

Analysis of confound predicted features: I do not understand the usage of features predicted by the confound as an analysis step. As far as I understand it, these are just a deterministic function of the confound, so I do not see any additional information that could be obtained by examining the confounded predicted features and their relationship to the outcome instead of confounds themself.

Response R. 1 C. 4:

Thank you very much for this insightful comment.
Indeed our claims regarding the use of confound predicted features (X_hat) were too strong. The idea we wanted to explore was to investigate the variance explained by the confounds that is removed from the feature. When there is an increase in performance after CR, the two possibilities are either suppression or leakage. In case of suppression, X_hat has to be either noise or a weak association on which the previous prediction given X was based upon. Therefore, we proposed that X_hat should not be more predictive than X itself in case of suppression/revealed information. But as the reviewer mentions, X_hat is a linear combination of the confounds. Thus, we agree with the reviewer that for TaCo it is not beneficial to look at X_hat. Therefore, we have removed all claims about X_hat from the manuscript and only rely on shuffled features to indicate confound-leakage.

To address this comment we have modified the following sentences:

Section: Walk-through analysis (page 3):
“””
~~and more importantly confound-predicted-features (^X) is higher than the baseline performance using original features (X).~~
“””

“””

~~Nevertheless, it can be argued that confound-leakage on the shuffled features, does not necessarily imply leakage for the non-shuffled features. Therefore, we used confound-predicted features ^X to gain direct evidence for confound-leakage using the non-shuffled features. In case of information-reveal, an increase in prediction performance after CR is due to removal of noise or weakly informative variance such as linear shortcuts. This means that the confound-predicted features ^X can only be predicting this weakly/ not informative variance in fact meaning that ^X can only be at most as predictive as X. In other words, higher accuracy when using ^X than X provides evidence of confound-leakage. In this walk-through example ^X (AUCROC = 1.00) achieved higher prediction score than X (AUCROC = 0.75) providing direct evidence of confound-leakage. Together shuffling the features and ^X-based prediction clearly demonstrate that the prediction boost is due to confound-leakage rather than information-reveal.~~

“””

Section: “CR using weaker confounds also increases performance”, page 4

“””

~~Inline with these results, ^X was also able to predict the target better than X (Fig. , Supplementary Fig. S1)~~

…

~~and 3/10 where ^X had performed better than X~~

“””

Section: “Confound-leakage poses danger in clinical applications”, page 5

“””

To disentangle the effect of each confound, we looked at the performance after CR for each confound separately.

Performing CR with BDI led to a high AUCROC with original features after CR (M = 0.91, SD = 0.01), shuffled features (M = 0.84, SD = 0.01) ~~and ^X (M = 0.84, SD = 0.01).~~

“””

Section: “Discussion”, page 5

“””

Specifically, by comparing the without CR baseline performance with CR after feature shuffling (X̃CR) ~~and features as predicted by the confound (^X)~~, this framework can identify confound-leakage as the cause of increased predictive performance.

“””

Section: “Discussion”, page 6

“””

~~For more direct evidence, the predictive performance of the confound predicted features (^X) can be assessed.~~

“””

Furthermore we removed the method section where we explain X_hat as measurement:
“”””

~~Predictability of ^X~~

~~Whenever CR lead to an increase in performance this can only have one of two reasons: either 1) revealing information present in the features, or 2) leaking confounding information. To reveal information in the features the CR has to suppress variance in the features which make learning generalizable features-target relationship harder. For example, unrelated noise or linear shortcuts could be suppressed. In other words, suppression works by removing less predictable variance in the data. This means that ^X has to be less predictive of the target than X in the resulting CR-ML workflow. If one finds contrasting evidence, an especially highly predictive ^X, this is strong direct evidence for confound-leakage through CR~~
“”””

Lastly, we relied on X_hat for the second walk-through analysis (result section page 3) which we have now adjusted accordingly to not include X_hat as evidence:

“”””

Confound removal for regression

As an example of a weaker confound on a regression task, we simulated a binary confound and then sampled a feature from different distributions for each confound value (confound equal to 0 or 1).

Then we added the confound to a normally distributed target (M = 0 and SD = 0.50 , Fig. 1 e-f). This creates a clear confounding situation, where the confound affects both the feature (Point-biserial correlation = 0.71, $p < 0.01$) and the target (Point-biserial correlation = 0.71, $p < 0.01$) and thus leads to a spurious relationship between the feature and the target (Pearson’s correlation = 0.51 , $p < 0.01$).

Following the same procedure as in the previous example, we observed increased performance after CR using a DT with limited depth of two (R2 using X = 0.29 , XCR = 0.42). As in this simulated data only a spurious relation (via confound) exists between the feature and target, it is safe to assume that an increased performance after CR is due to confound-leakage. ~~Still, shuffled features were not sensitive to confound-leakage (X̃ = 0 , X̃CR = –0.01). On the other hand, ^X-based predictions clearly indicate confound-leakage (^X = 0.51).~~ Furthermore, we found a probable mechanism behind this confound-leakage to be the distribution of the features conditioned on the confound. More precisely, CR shifts the feature values for confound = 1 in between most feature values for the confound = 0 (Fig. 1 e). This leaks the confounding information into the feature instead of removing it (Fig. 1 f). The shuffled features, however, were not sensitive to confound-leakage (X̃ = 0 , X̃CR = –0.01), which is expected considering the probable cause for such leakage depends on the joint distribution of the confound and the feature. When shuffling the features within each confound category to preserve the joint distribution, we observed an increase in performance after CR (M=0.29 before to M=0.42). This result indicates that shuffling the features might not be always sensitive to confound-leakage. We, nevertheless, use independently shuffled features in our analysis for practicality, particularly in the context of continuous or multiple confounding factors.
“”””

We have removed all the X_hat from the plots as well. See end of this document for an overview of all figures.

R. 1 C. 5.:
References: I think that our paper [1] should be referred to more extensively. We did not describe the problems described in this manuscript, but our findings are relevant to his paper and will also interest readers. Specifically:

Response R. 1 C. 5:
Thank you very much for your comment. The paper [1] is indeed of interest and shows several relevant analyses. However, there is a key difference between the problems described in [1] and our work. While [1] deals with measuring residual confounding after removal, we address the introduction of confounding through confound removal. This is why we are hesitant to make too many direct connections between these two different questions. Still, we understand that one could discuss the relevance of your paper more. Below we address each sub comment separately.

Subcomment a. We have extensively discussed the pitfalls of confound removal. This should be mentioned in the introduction and discussion when discussing the confound removal. Consider discussing these pitfalls in a few sentences. In light of our paper, instead of framing confound regression as something that is expected to work, it should be framed as something that was already reported to be problematic.

Response a:
Thank you for your remark. We have added the following sentences to highlight that CR is already seen as problematic due the pitfall of leaving residual confounding information after removal.

- *Introduction (page 2)*:
“”””
It is important to note that CR is not without other pitfalls, for instance it might fail to completely remove confounding information [21, 27]. Still, CR is considered the de facto method, and therefore analyzing the hitherto unknown pitfall of leaking confounding information through CR is helpful. Furthermore, there were speculations of ~~Although a recent study has speculated~~ confound-leakage in ML workflows [18], it has not yet been systematically shown, analyzed nor explained.
“”””

- *Discussion (page 5):*
“”””
Specifically, we have shown this method can counter-intuitively introduce confounding, which can be exploited by some non-linear ML algorithms.
Thus in addition to the already known pitfalls of residual confounding [21], our results show that CR may actually introduce confounding-information.
“”””

Subcomment b:

The explanations in the section "confound-leakage due to deviation from normal distributions," including figures 1e and 1f, is related to our explanations of why confound removal does not sufficiently clean data from confounding information, and I think this should be mentioned.

Response b:
We have added the following sentence to the Results (page 4) in response to this request:
“”””
These simulations show that skewed features, and by extension potentially other non-normal distributed features, can lead to confound-leakage. Interestingly, another consequence of non-normal distributions is insufficient removal of confounding information [21].
“”””

Subcomment c:
Using a target as a confound to test the validity/reliability of confound removal was also done in our paper, and I think it should be mentioned.

Response c:
We have modified the text in response to this request (Introduction, page 3):
“”””
Indeed, as we show, the TaCo framework reveals strong effects where the prediction accuracy is boosted from moderate to perfect as well as weaker effects for confounds weakly correlated with the target. A previous work has used TaCo for evaluating the validity and reliability of confound adjustment methods [21].
“”””

Subcomment d:
The method to deal with confounding presented in our paper will guard against the dangers of confound regression described in this manuscript. Our method is based on estimating what variance in the outcome can be explained using ML predictions that confounds cannot explain, so in the target as confound situation, this will be 0, thus the dangers of confounded results should be avoided. This should be discussed more in the recommendation section. Also, statements like "we made progress on understanding these issues, there is no full-proof method for detecting and eliminating leakage," and "we are not aware of a procedure to definitively exclude confound-leakage as an explanation." etc. should be modified since I believe that our method, although not perfect, is a candidate solution for this problem and it would of interest to readers to offer them a solution, of course with caveats. Another solution not discussed in the manuscript would be the permutation test mentioned in point 3.

Response d:
Thank you very much for your comment. In fact, we have already mentioned your work as well as permutation testing in the Discussion (page 6).

“”””
Other methods can be employed, e.g., proposed by Spisak [27]. Furthermore, measuring how dependent the predictions of a model are on the confound by permutation testing [34, 35] or the approach proposed by Dinga et al. [21] can be helpful.
“”””

To further emphasize the importance the methods mentioned by you we have added them to the next recommendation section:
“”””

To gain additional information, the reader might be interested in methods to estimate the variance in the target explained by ML predictions that confounds cannot explain [21, 27].
“”

We think it is important to not make claims of any full-proof methods. As mentioned in the new section mentioned in *Response R. 1 C. 3*, comparing models trained in different scenarios can be problematic as similar performance can be achieved by relying on different types of information both present in the data, e.g. a combination of suppression and leakage. As the reviewer has pointed out that their method is not without caveats, we do not feel comfortable to claim that any of the discussed candidates are full-proof.

R. 1 C. 6:
High-dimensional data are more susceptible to confound leakage: I am not entirely sure this claim is correct. For me, intuitively, it is not. I can imagine that biases created within individual features by confound removal might average out to 0 in high-dimensional data in the test set. I think it would be worth performing a controlled simulation to support this claim.

Response R. 1 C. 6:
Thank you very much for your feedback.
We had already included a controlled simulation to support this claim in the manuscript, but we agree that it was not featured prominently enough. To address this we have now moved an adjusted version of the former Supplementary Fig. 2 into the manuscript as Fig. 5 and added a new simulation to further support this claim (Results, page 4).

“”
Lastly, we investigated whether such effects could also occur when randomly sampling non-normal distributed features instead of carefully constructing the features conditioned on the confound. To this end, we sampled an increasing number of features (1 to 100) either using a random normal or skewed (χ2, df = 3) distribution independent of a normally distributed target.
Using RF, we observed increased performance after TaCo removal with skewed features but not with normally distributed features, e.g. R2 of M = 0.23 with SD = 0.06 compared to R2 of M = –0.04 with SD = 0.04, respectively with 100 features. Importantly, this effect increased with the number of features (~~Supplementary Fig. S2~~ Fig 5).
To further illustrate this point, we performed another simulation depicting a typical confounding situation. Here, we sampled an increasing number of features (1 to 100) with different χ2 distribution given a binary confound (df=3 (4) and scale=0.5 (1) for confound=0 (1)). The target was sampled from a normal distribution (M=0, SD=0.2) and the confound was added to it. Analysis of this data shows an increased performance after confound removal from M=-0.52 (SD=0.02) to M=-0.50 (SD=0.03) using one feature and from M=-0.02 (SD=0.01) to M=0.18 (SD=0.01) using 100 features. These results demonstrate that the effect of confound-leakage increases with increasing number of features.
“”

Minor

7. I think the methods used need a little more explanation, and the structure could be a little improved. Although what "target as confound" does is, in a way, self-evident, the procedure and motivation for it

can be explained a bit more. Also, now the whole explanation is at the end of the introduction section, but it should be elsewhere.

Thank you very much for this excellent point. We agree that a more detailed methods section will improve the structure and readability. We still would like to keep the explanation of TaCo in the introduction to guide readers through the paper.
We have added the following subsection to the Methods:

“”

Target as a Confound (TaCo)

The TaCo framework allows systematic analysis of confound removal effects. Confounding is a three-way relationship between features, confounds and the target. This means that a confound needs to share variance with both the feature and the target. Measuring or simulating such relationships can be hard especially if linear univariate relationships cannot be assumed. Furthermore, effects of confound removal should increase with the actual strength of the confound. The target itself explains all the shared variance and thus it is the strongest possible confound. Therefore, using the target as a confound, i.e. TaCo, measures the most possible extent of confounding. In addition, using the TaCo simplifies the analysis to a two-way relationship. Lastly, the TaCo approach is applicable to any dataset and can help to measure the strongest possible extent of confound-leakage even without knowing the confounds.
“”

8. possible bug: I suspect that in your walkthrough analysis, X_train_shuffled variable is not shuffled since you are calling X_train_shuffled = pd.DataFrame(zscore.transform(X_train), columns=col_names[:-1])
and not X_train_shuffled = pd.DataFrame(np.random.permutation(zscore.transform(X_train)), columns=col_names[:-1]) as in other variables. But I am not an expert on the workings of the code. Nevertheless, it won't change anything about the paper. The biases described by the authors will still be present.

Thank you very much for looking at the code and finding this bug!
Indeed, this was a bug that we have fixed. As you said, it makes no difference for the interpretation of our results. The following values changed in a non fundamental way due to this bug-fix (Walk-through analysis, page 3):

“”

We observed chance-level performance without CR (AUCROC = ~~0.52~~0.48) for the shuffled features. However, a performance increase after TaCo removal was observed (AUCROC = ~~0.98~~0.99). This analysis shows that performance increase after TaCo removal with shuffled features indicate the possibility of confound-leakage.
“”

Reviewer 2 (R.2.):
The manuscript overrides the common perception that linear regression can help remove confounding effect in machine learning analysis. Experiments showed that in certain scenarios linear regression can inject confounding effects that can be picked up by non-linear machine learning models, e.g., by achieving higher prediction accuracy even when using shuffled features. Two hypotheses are given why such phenomenon can happen.

I think some part of the analysis resolved my long-lasting confusion in my own studies. Interesting observations overall, but there are three major concerns that I hope the authors can respond to:

We thank you for your encouraging words.

R. 2 C. 1.
While linear regression is a standard way to remove confounding effects, how to construct that regression can vary from case to case. In the scenario where the confounder is highly correlated with the label, people sometimes incorporate both label and confounder as covariates, or apply linear regression only in the control group. The idea is that you need to disentangle variance in the feature explained by the label from the variance explained by the confound. Again, feature, label, and confound have three-way dependencies by definition and failure to discuss this aspect can be misleading.

Thank you very much for your insightful comment.
First, indeed, adding the confounders as covariates to the model is a common approach in statistical analysis which can shed light on how much variance is explained by the confounder. However, the focus of this work is building ML models that do not use confounding information, i.e. they are confound-free. Therefore such ML set-ups do not include confounds as features. To clarify our focus, we have added the following text to the Introduction (page 2):

“”””

Two methods for treating confounding are commonly employed in data analysis with the goal of building an accurate ML model that is not biased by the confounding information.
“”””

Second, we thank you for mentioning the possibility of training confound removal models only on the control group. We have incorporated this set-up into the manuscript by repeating our analysis for the clinically relevant ADHD dataset while training CR only on the healthy group and then removing their variance from the data as described by you and Dukart et al. [2]. The results confirm our previous statements as we observe the same pattern of increase in accuracy due to confound leakage as using standard CR. To highlight this point we added the following sentences to the Results (subsection: “Confound-leakage poses danger in clinical applications”, page 5):

“”””

Training CR models only on healthy individuals can be helpful in clinical applications [4]. We investigated this variant of CR and again the AUCROC increased for original features after CR M=0.83 (SD=0.02) and an increase with shuffled features from M=0.51 (SD=0.05) to M=0.79 (SD=0.02), suggesting that confound leakage is also a concern for variants of CR.
“”””

Also thank you very much for the remark that readers should always consider the actual problem to be a three-way depency. This complexity is one of our key motivations of using TaCo and therefore very important for this paper. Therefore we highlight this in our new method section:

“”””

Target as a Confound (TaCo)

The TaCo framework allows systematic analysis of confound removal effects. Confounding is a three-way relationship between features, confounds and the target. This means that a confound needs to share variance with both the feature and the target. Measuring or simulating such relationships can be hard especially if linear univariate relationships cannot be assumed. Furthermore, effects of confound removal should increase with the actual strength of the confound. The target itself explains all the shared variance and thus it is the strongest possible confound. Therefore, using the target as a confound, i.e. TaCo, measures the most possible extent of confounding. In addition, using the TaCo simplifies the analysis to a two-way relationship. Lastly, the TaCo approach is applicable to any dataset and can help to measure the strongest possible extent of confound-leakage even without knowing the confounds.
“”””

R. 2 C. 2.
I generally do not agree with the claim that "\hat{X} can only be at most as predictive as X", especially in the TaCo setting. Using label as the super confound, we are basically trying to explicitly preserve label-related component in the feature and discard information irrelevant to the label. This actually makes the classification simpler, which does not indicate confounder-leakage. If authors agree with my view, I would suggest changing their interpretation of this part of the results or removing them.

Thank you very much for your remark.
Your feedback and the comment 4 from Reviewer 1 have led us to rethink the interpretation of X_hat. We acknowledge that we overinterpreted its usefulness and have now removed it from the manuscript as it does not provide more evidence than just predicting the target from the confound. We now only rely on the shuffling approach which we have also extended for clinically relevant dataset to include a permutation test.

To address this comment we have modified the following sentences:

Section: Walk-through analysis (page 3):
“”””
~~and more importantly confound-predicted-features (^X) is higher than the baseline performance using original features (X).~~
“”””

“”””
~~Nevertheless, it can be argued that confound-leakage on the shuffled features, does not necessarily imply leakage for the non-shuffled features. Therefore, we used confound-predicted features ^X to gain direct evidence for confound-leakage using the non-shuffled features. In case of information-reveal, an increase in prediction performance after CR is due to removal of noise or weakly informative variance such as linear shortcuts. This means that the confound-predicted features ^X can only be predicting this weakly/ not informative variance in fact meaning that ^X can only be at most as predictive as X. In other words, higher accuracy when using ^X than X provides evidence of confound-leakage. In this walk-through example ^X (AUCROC = 1.00) achieved higher prediction score than X (AUCROC = 0.75) providing direct evidence of confound-leakage. Together shuffling the features and ^X-based prediction clearly demonstrate that the prediction boost is due to confound-leakage rather than information-reveal.~~
“”””

Section: “CR using weaker confounds also increases performance”, page 4
“”””
~~Inline with these results, ^X was also able to predict the target better than X (Fig. , Supplementary Fig. S1)~~
…
~~and 3/10 where ^X had performed better than X~~
“”””

Section: “Confound-leakage poses danger in clinical applications”, page 5
“”””
To disentangle the effect of each confound, we looked at the performance after CR for each confound separately.
Performing CR with BDI led to a high AUCROC with original features after CR (M = 0.91, SD = 0.01), shuffled features (M = 0.84, SD = 0.01) ~~and ^X (M = 0.84, SD = 0.01).~~
“”””

Section: “Discussion”, page 5
“”””
Specifically, by comparing the without CR baseline performance with CR after feature shuffling (X̌CR) ~~and features as predicted by the confound (^X)~~, this framework can identify confound-leakage as the cause of increased predictive performance.
“”””

Section: “Discussion”, page 6
“”””
~~For more direct evidence, the predictive performance of the confound predicted features (^X) can be assessed.~~
“”””

Furthermore we removed the method section where we explain X_hat as measurement:
“”””

~~Predictability of ^X~~

~~Whenever CR lead to an increase in performance this can only have one of two reasons: either 1) revealing information present in the features, or 2) leaking confounding information. To reveal information in the features the CR has to suppress variance in the features which make learning generalizable features-target relationship harder. For example, unrelated noise or linear shortcuts could be suppressed. In other words, suppression works by removing less predictable variance in the data. This means that ^X has to be less predictive of the target than X in the resulting CR-ML workflow. If one finds contrasting evidence, an especially highly predictive ^X, this is strong direct evidence for confound-leakage through CR~~
“”””

Lastly, we relied on X_hat for the second walk-through analysis (result section page 3) which we have now adjusted accordingly to not include X_hat as evidence:

“”””

Confound removal for regression
As an example of a weaker confound on a regression task, we simulated a binary confound and then sampled a feature from different distributions for each confound value (confound equal to 0 or 1).
Then we added the confound to a normally distributed target (M = 0 and SD = 0.50 , Fig. 1 e-f). This creates a clear confounding situation, where the confound affects both the feature (Point-biserial correlation = 0.71, $p < 0.01$) and the target (Point-biserial correlation = 0.71, $p < 0.01$) and thus leads to a spurious relationship between the feature and the target (Pearson’s correlation = 0.51 , $p < 0.01$).
Following the same procedure as in the previous example, we observed increased performance after CR using a DT with limited depth of two (R2 using X = 0.29 , XCR = 0.42). As in this simulated data only a spurious relation (via confound) exists between the feature and target, it is safe to assume that an increased performance after CR is due to confound-leakage. ~~Still, shuffled features were not sensitive to confound-leakage (X̆ = 0 , X̆CR = –0.01). On the other hand, ^X-based predictions clearly indicate confound-leakage (^X = 0.51).~~ Furthermore, we found a probable mechanism behind this confound-leakage to be the distribution of the features conditioned on the confound. More precisely, CR shifts the feature values for confound = 1 in between most feature values for the confound = 0 (Fig. 1 e). This leaks the confounding information into the feature instead of removing it (Fig. 1 f). The shuffled features, however, were not sensitive to confound-leakage (X̆ = 0 , X̆CR = –0.01), which is expected considering the probable cause for such leakage depends on the joint distribution of the confound and the feature. When shuffling the features within each confound category to preserve the joint distribution, we observed an increase in performance after CR (M=0.29 before to M=0.42). This result indicates that shuffling the features might not be always sensitive to confound-leakage. We, nevertheless, use independently shuffled features in our analysis for practicality, particularly in the context of continuous or multiple confounding factors.
“”””

We have removed all the X_hat from the plots as well. See end of this document for an overview of all figures.

R. 2 C. 3.
Most of the analyses were based on simulation. Most of the interpretation was based on accuracy scores. What is more interesting is the mechanism why it happens in real applications. The authors have two hypotheses, which I believe are not that hard to test on the real data set. One can look at features that drive the X_CR classification but not the raw classification, and then look at how their distribution changes with respect to confounders. One can probably visualize the distribution shift over multi-dimensional features using certain dimension reduction techniques. With those results, the manuscript will become more valid.

Thank you very much for this important point. We agree that it is very interesting to look into the real world data and check whether our clinical data has confound-leakage due to deviation from normal distributions. To this end, we followed your advice and looked into the feature importance and visualized the most important features with and without confound removal.
We have added four new panels to Fig. 4 (shown below) showing feature importance differences before and after CR and how the two most important features lead to leakage. Furthermore, we added the following text to the Results section (page 5):

“”””

Lastly, we wanted to evaluate why we observe confound-leakage on this dataset. The limited precision of features cannot be the reason here as all features are continuous. Therefore, we hypothesized that the confound leaked due to some features deviating from normal distributions. To this end we first compared the feature importance between the RF after CR and using the original features. Here, we observed the RFs’ 10 most important features were completely different (Fig. 4 c-d), indicating that the two RF models rely on different relationships in the data. Next we visualized the distributions of the two most important features of the RF after CR for both models. This visualization (Fig. 4 e-f) clearly shows that CR has shifted the distributions due to deviations from normal distributions leaking information in their joint distribution. Furthermore, we trained new DTs using only these two features before or after CR. This led to an increase of AUCROC from 0.61 to 0.70 after CR only using these features.
These analyses clearly demonstrate that real-world applications could suffer from confound-leakage and users should exercise care when implementing and validating a CR-ML workflow.
“”””

We did not use a dimensionality reduction to not complicate our methods. We have modified figure 4 (shown below) to include the ten most important features before and after CR. Notably, the two models highlight different features. Next we also visualized the two most important features after CR and show that one can indeed observe a shift of distributions

leaking information through CR.

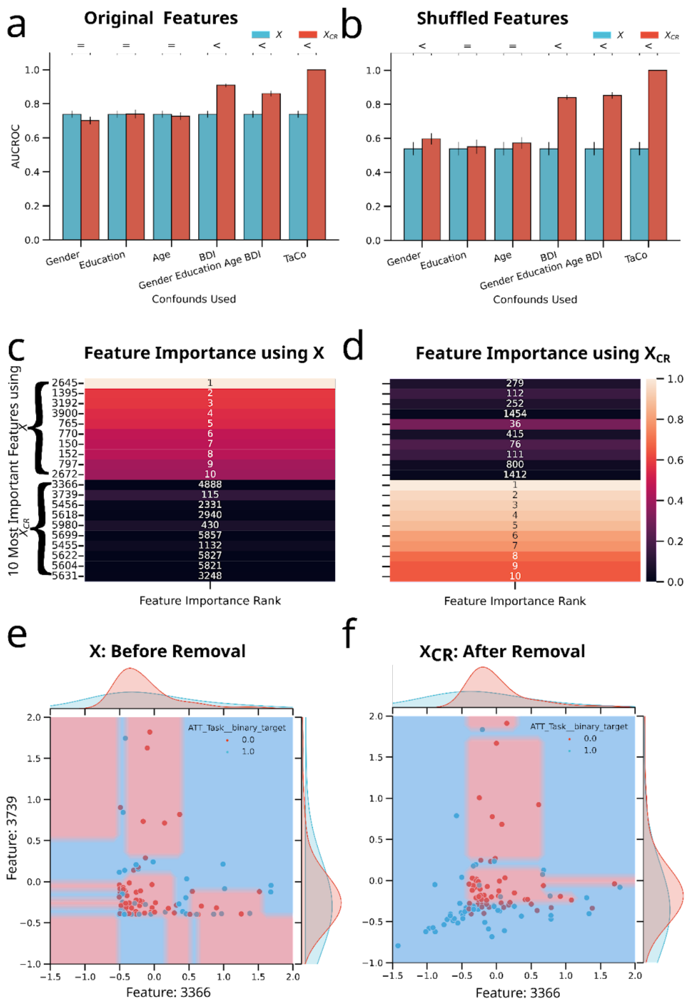

We would like to note that simulations allow us to control relationships between features, confounds and target, which is needed to really show the extent of confound-leakage and elucidate its possible mechanisms.

Minor: figure captions are generally terse. Axis labels are confusing, e.g., Figure 1c,d. In figure 2, why are there two rows of r2 and two rows of AUC? What do you mean by those four rows of 'score'? Do they belong to the right column? Do < and > indicate statistical significance? People often use *, ** to indicate p<0.05, p<0.005, because the direction is obvious from the plots already.

Thank you very much for highlighting the problem that our statistical analyses are not communicated clear enough. We have updated the figures and have added more details to the methods section to address your comments.

The statistical tests we employed are not frequentist null hypothesis testing. Instead we used the Bayesian ROPE approach for the following reasons:

- Assumption of no difference in performance metrics (scores) rarely makes sense in ML settings.

- Significance cannot compute the probabilities of interest: Probability of one pipeline scoring higher than the other one.
- Significance tests are highly dependent on the sample size.

More details can be found in the paper that introduced this approach [3].

We added the following section to our methods to communicate this to the reader:
“””

The Bayesian ROPE for model comparison

In this study we used the Bayesian ROPE [42] approach to qualify differences between K-fold cross-validation results coming from two models. This approach uses the Bayesian framework to compute probabilities of the metric falling into a defined region of practical equivalence or of one ML pipeline scoring higher than the other. This is achieved by defining a region of equivalence (here we used 0.05). Consequently, the Bayesian ROPE approach allows us to make probabilistic statements regarding whether and if so which of the ML pipelines score higher. We summarize these differences using the following symbols = (highest probability of pipelines scoring practically equivalent), < (highest probability of right pipeline scoring higher), > (highest probability of left pipeline scoring higher). Other possibilities such as the significance test correcting for the dependency structure in K-fold CV [43] or permutation testing by shuffling the target or features can be employed when suitable.“””

We have adjusted the figure captions as shown below by providing additional information making them more self sufficient.

Adjusted Figures Overview

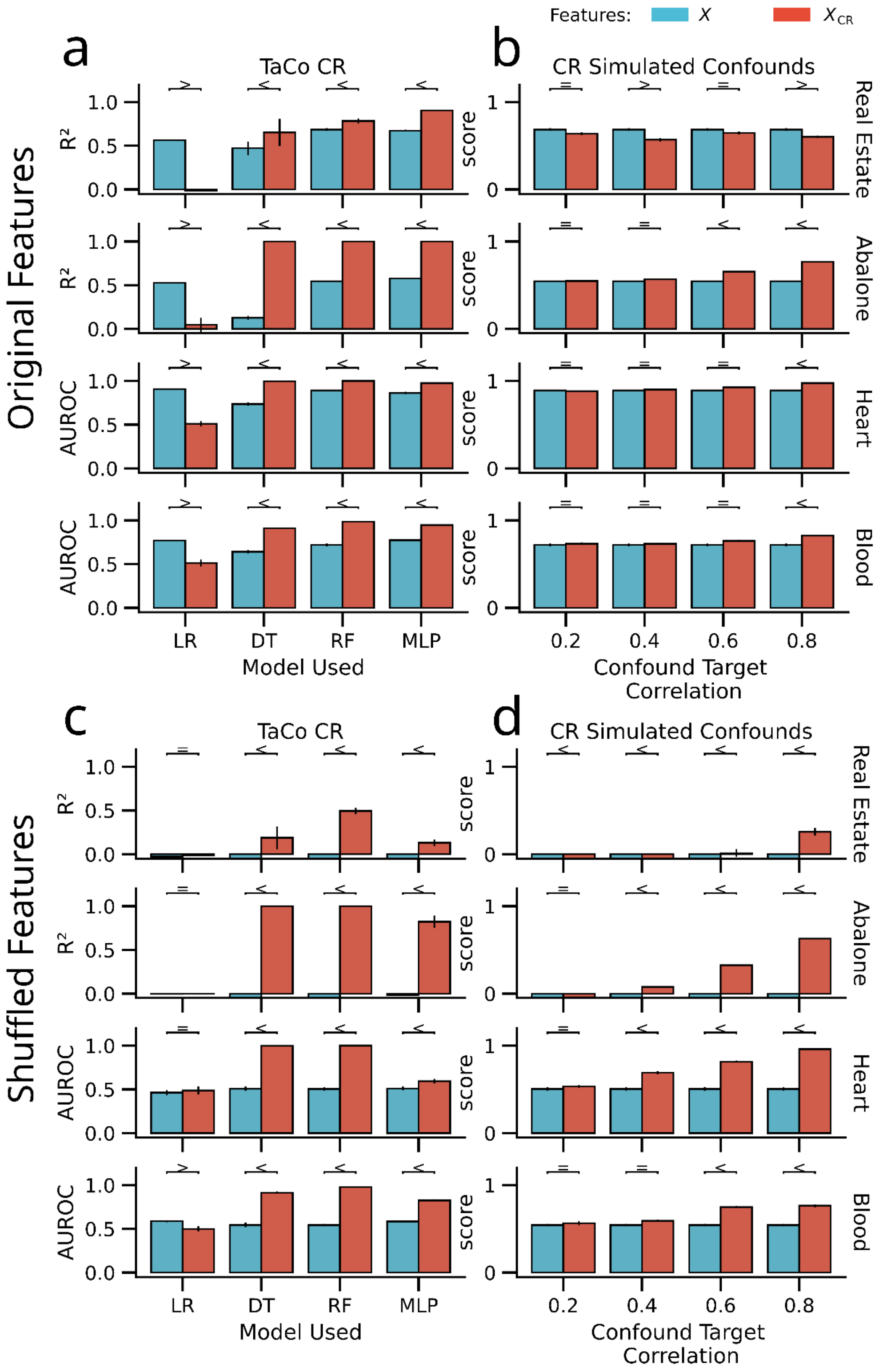

“”

Figure 2. Performance on the UCI benchmark datasets when using raw vs CR features (a) and raw vs the predicted features given the confound/TaCo/~~^X~~ (b). The two columns correspond to: 1) TaCo removal with four ML algorithms (LR, DT, RF, MLP), and 2) CR with simulated confound with different correlation to the target (range 0.2-0.8) with RF. (a,b) show performance using the original features while (c,d) show the performance on shuffled features.
To check whether a difference between the performance of two models is meaningful, we used the Bayesian ROPE approach to identify what is most probable: performance being higher before removal (<), being higher after removal (>) or equivalent (=) (see the Methods section for details).
When using a linear model (LR) TaCo removal leads to reduction in prediction performance, as expected. In contrast, nonlinear models lead to a higher performance for all datasets. This increase could be either explained by confound removal revealing information already in the data (suppression) or confound removal leaking information into the features (confound-leakage). Shuffling the features destroys association between features and the target, therefore subsequent performance increase after TaCo removal indicates the possibility of confound-leakage (c,d). ~~Additionally, the higher performance of ^X (a,b) compared to X does not support suppression as explanation as suppression assumes that confound-removal removes noise or other at most weakly predictive variance from the features. In this case, the variance removed feature ^X should be less predictive than the raw features X.~~ The simulated confounds show that an increase after CR is also possible for confounds weakly related to the target (b,d) and one dataset (Blood) shows strong evidence of confound-leakage.
“”

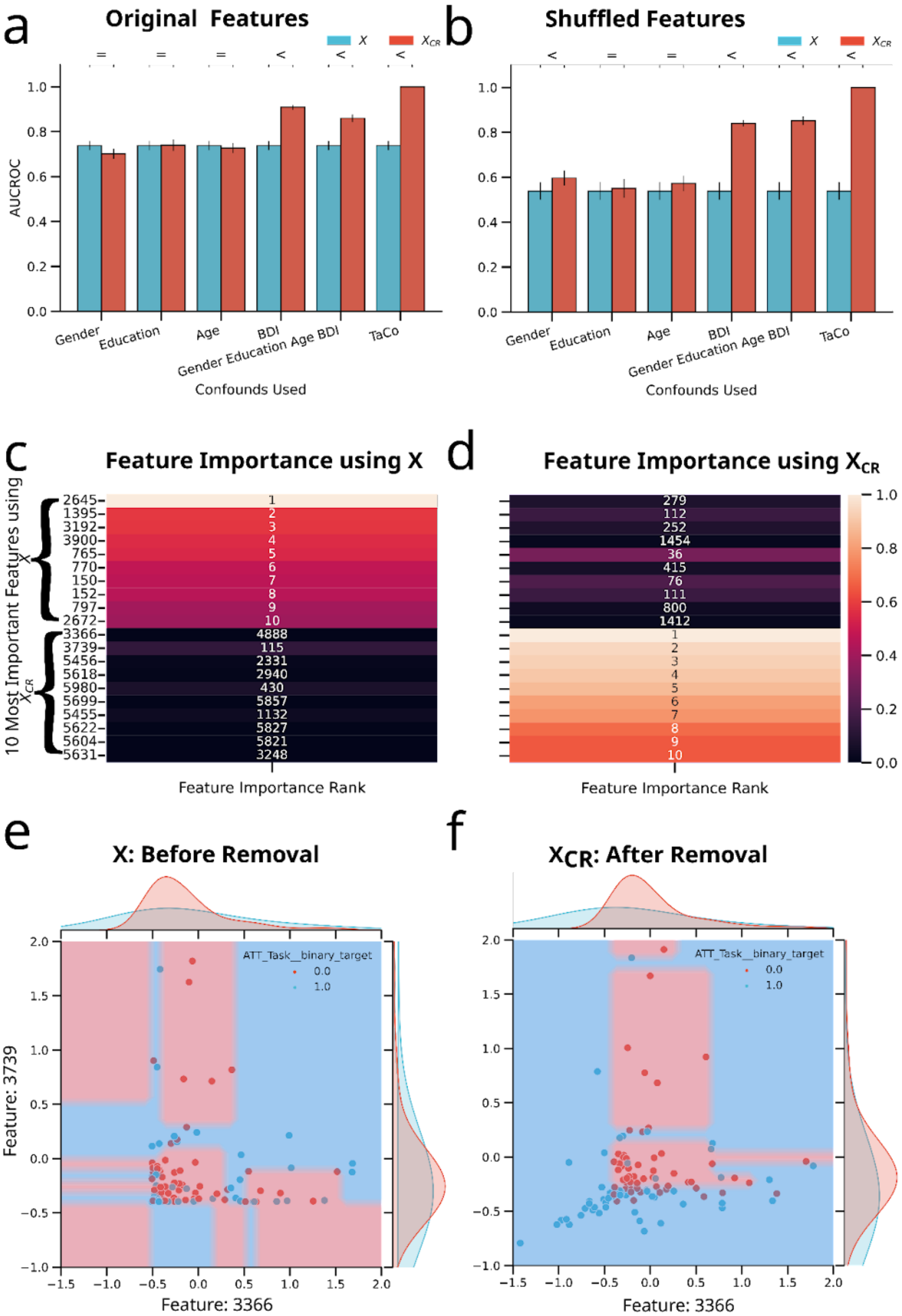
a
Original Features
b
Shuffled Features
X
X_CR
AUCROC
Gender
Education
Age
BDI
Gender Education Age BDI
TaCo
Confounds Used
c
Feature Importance using X
d
Feature Importance using X_CR
10 Most Important Features using X
X_CR
Feature Importance Rank
e
X: Before Removal
f
X_CR: After Removal
ATT_Task__binary_target
Feature: 3739
Feature: 3366

“”””

Figure 4. ~~Summary of the performance on t~~The real-world ADHD speech dataset. The performance when using different confounds (a-b), most important features of RF when using BDI as confound (c-d) and visualization of confound-leakage due to deviation from normal distributions (e-f).

~~Note that the features used were always the same.~~

~~Increased performance, for both original and shuffled(features, can be seen when using the TaCo and when BDI was used as a confound. This suggests that BDI is driving the performance increase~~

a shows the performance of a RF predicting ADHD vs healthy controls using the original features. To check whether a difference is meaningful we used the Bayesian ROPE approach to identify what is most probable: performance being higher before removal (<), being higher after removal (>) or equivalent (=) (see method section). An increased performance can be observed when using all confounds, BDI as a confound or the TaCo. The same pattern appears when the features were shuffled (b). This shows that the increase in performance is due to confound-leakage and BDI is a driving factor for this leakage as it leaks information when used as a confound. c-d visualize the 10 most important features for both using X and X_CR as features. The feature ranking is shown as white label on top of each cell. The most important features are different for X and X_CR. Furthermore, the most important features of one model ranked as very unimportant in the other. e-f show decision boundaries of DT trained on the two most important features after CR. The background colors indicate the prediction of the model, the points show the true target value and the x-axis the two most important features. The distribution of each feature conditioned on the target is shown as the density plots. One can see that CR leaks information by cleanly separating the blue and red points.

“”””

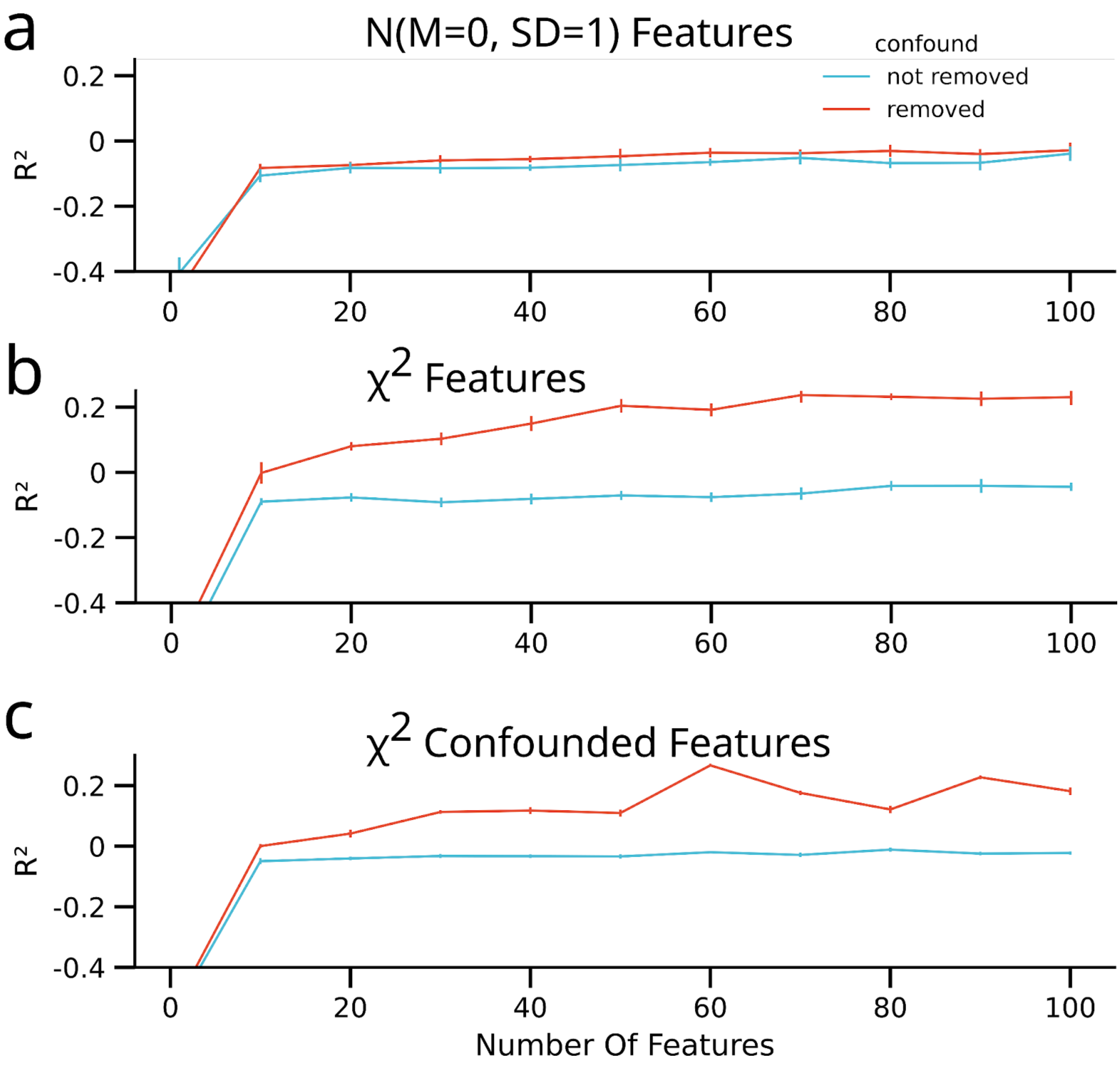

Fig. 5. Prediction performance of a RF trained with (blue) or without (red) confound removal on an increasing number of features. Each feature was either sampled from a random standard normal distribution (mean=0, std=1), a random χ2 distribution with df = 3 or a χ2 distribution with a df=3, scale=0.5 or df=4, scale=1 for the confound being equal to 0 and 1 respectively. a) The RF trained on the normally distributed features did not achieve performance above the chance level (R2 < 0) irrespective of confound removal. b-c) When training the RF on either of the χ2 distributed features, confound removal resulted in above chance level performance (R2 > 0). This effect increased with an increasing number of features and can only be explained by confound removal leaking information into the features.

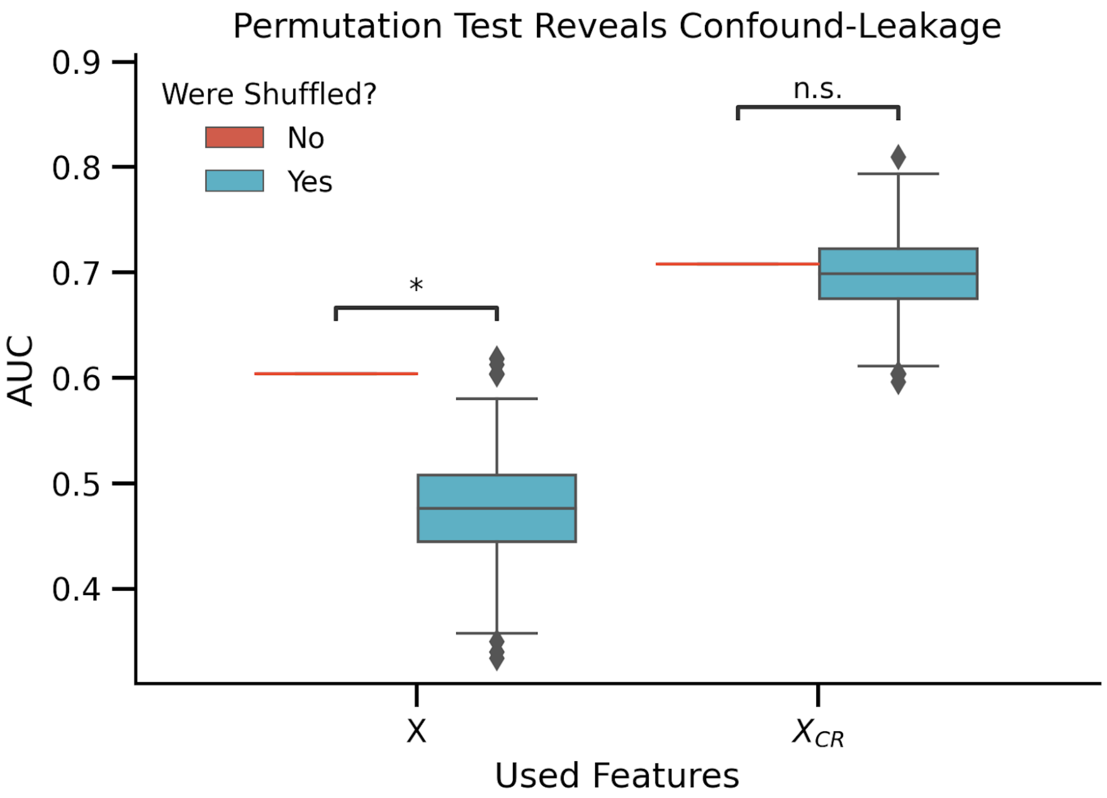

Fig. S2. We performed permutation testing with 1000 iterations. After shuffling the features, a significantly lower performance was observed compared to the original features X. No significant difference between raw and shuffled features was observed when using the X_CR features. This result is in line with the leakage hypothesis as the higher accuracy after shuffling and CR indicates leaking target-related confounding information into the features.

References Response Letter:

[1] Dinga R, Schmaal L, Penninx BWJH, Veltman DJ, Marquand AF. Controlling for effects of confounding variables on machine learning predictions. bioRxiv 2020;

[2] Dukart J, Schroeter ML, Mueller K. Age correction in Dementia - Matching to a healthy brain. PLoS ONE 2011;6.

[3] Benavoli A, Corani G, Demšar J, Zaffalon M. Time for a change: A tutorial for comparing multiple classifiers through Bayesian analysis. Journal of Machine Learning Research 2017;18
