# Supplementary material for: Confound-leakage: confound removal in machine learning leads to leakage
Source: Gigascience. 2023 Sep 30;12:giad071. doi: 10.1093/gigascience/giad071 (PMC10541796; doi:10.1093/gigascience/giad071)
Supplement: giad071_Supplemental_File [file giad071_supplemental_file.pdf]

Supplementary Information

Confound-leakage: Confound Removal in Machine Learning Leads to Leakage

Sami Hamdan[1,2], Bradley C. Love[3,4,5], Georg G. von Polier[1,6,7], Susanne Weis[1,2], Holger Schwender[8], Simon B. Eickhoff[1,2], Kaustubh R. Patil[1,2,*]

[1] *Institute of Neuroscience and Medicine,*
Brain and Behaviour (INM-7), Forschungszentrum Jülich, Jülich, Germany
[2] *Institute of Systems Neuroscience,*
Medical Faculty, Heinrich-Heine University Düsseldorf, Düsseldorf, Germany
[3] *Department of Experimental Psychology, University College London, London, UK*
[4] *The Alan Turing Institute, London, UK*
[5] *European Lab for Learning & Intelligent Systems (ELLIS)*
[6] *Department of Child and Adolescent Psychiatry,*
Psychosomatics and Psychotherapy, University Hospital Frankfurt, Frankfurt, Germany
[7] *Department of Child and Adolescent Psychiatry,*
Psychosomatics and Psychotherapy, RWTH Aachen University, Aachen, Germany
[8] *Institute of Mathematics,*
Heinrich-Heine University Düsseldorf, Düsseldorf, Germany

* *k.patil@fz-juelich.de*

Dataset	Problem Type	Sample Size (binarized)	Feature Numbers	Source
Income (Adult)	Classification	32561 (15682)	14	UCI
Bank Marketing	Classification	41188 (9280)	20	UCI
Heart	Classification	297 (274)	13	UCI
Blood Transfusion	Classification	748 (356)	4	UCI
Breast Cancer	Classification	569 (424)	10	UCI
Student Performance	Regression	649	30	UCI
Abalone	Regression	4177	8	UCI
Concrete Compressive Strength	Regression	1030	8	UCI
Residential Building	Regression	372	107	UCI
Real Estate	Regression	414	6	UCI
Speech ADHD	Classification	126	6016	no public access

Table S1. Overview of all the datasets used. Shows each dataset with their associated problem type, sample size, feature number and source. Our datasets cover a big range of features and sample sizes. All datasets with the exception of the speech ADHD one are freely accessible through the UCI machine learning repository.

Simulation	X	c	y	n
Walk-Through Continuous	where c==0: $\rightarrow N$(M=0,SD=0.5) where c==1: $\rightarrow concat($ N($M = -1, SD = 0.5$), N($M = -5, SD = 0.5$))	binary()	N($M = 0$, $SD = .5$) + c	2000
Weaker Confounds UCI Benchmarks UCI	Original UCI X	Pearson's r of $0.2 - 0.8$ with y	UCI	UCI
Deviation from normal distributions	concat(N($M = 0$,$SD = 1$, $n = 900$), where $c == 0$: $\rightarrow N$(M=-4,SD=.5,n=50), where $c == 1$: $\rightarrow N$(M=4,SD=.5,n=50))	=y	binary()	1000
Confound-leakage deviation from normal distributions	concat(N($M = 0$,$SD = 1$,$n = 900$), where c==0 $\rightarrow N$(M=-4,SD=.5,n=50), where c==0 $\rightarrow N$(M=4,SD=.5,n=50))	=y	binary()	1000
Limited Precision Perfect Balance	[[0, 1]*500]	=y	repeat($[0, 0, 1, 1]$, 250)	1000
Limited Precision Slight Unbalance	X from Limited Precision Perfect Balance swap_one(0 to 1, where $y == 1$) & swap_one(1 to 0, where $y == 1$)	=y	repeat($[0, 0, 1, 1]$, 250)	1000
Limited Precision Continuous No Rounding	N($M = 0$,$SD = 1$)	=y	N($M = 0$, $SD = 1$)	1000
Limited Precision Continuous Rounding	X from Limited Precision Continuous No Rounding round values to only vary in steps of 0.1	=y	N($M = 0$, $SD = 1$)	1000

Table S2. Overview of all the simulations used. Including pseudo code to create the features (X), target (y) and confounds (c). Variables were sampled from normal distributions (N), with different means (M) and standard deviations (SD) or binary distributions (binary). *repeat(list, number)* indicated the repetition of list of values ([value, value, ...]) are repeated for a number of times. *concat* means the concatenation of multiple arrays and *where condition* $\rightarrow$ *operation* means that the operation is executed for where the condition is met.

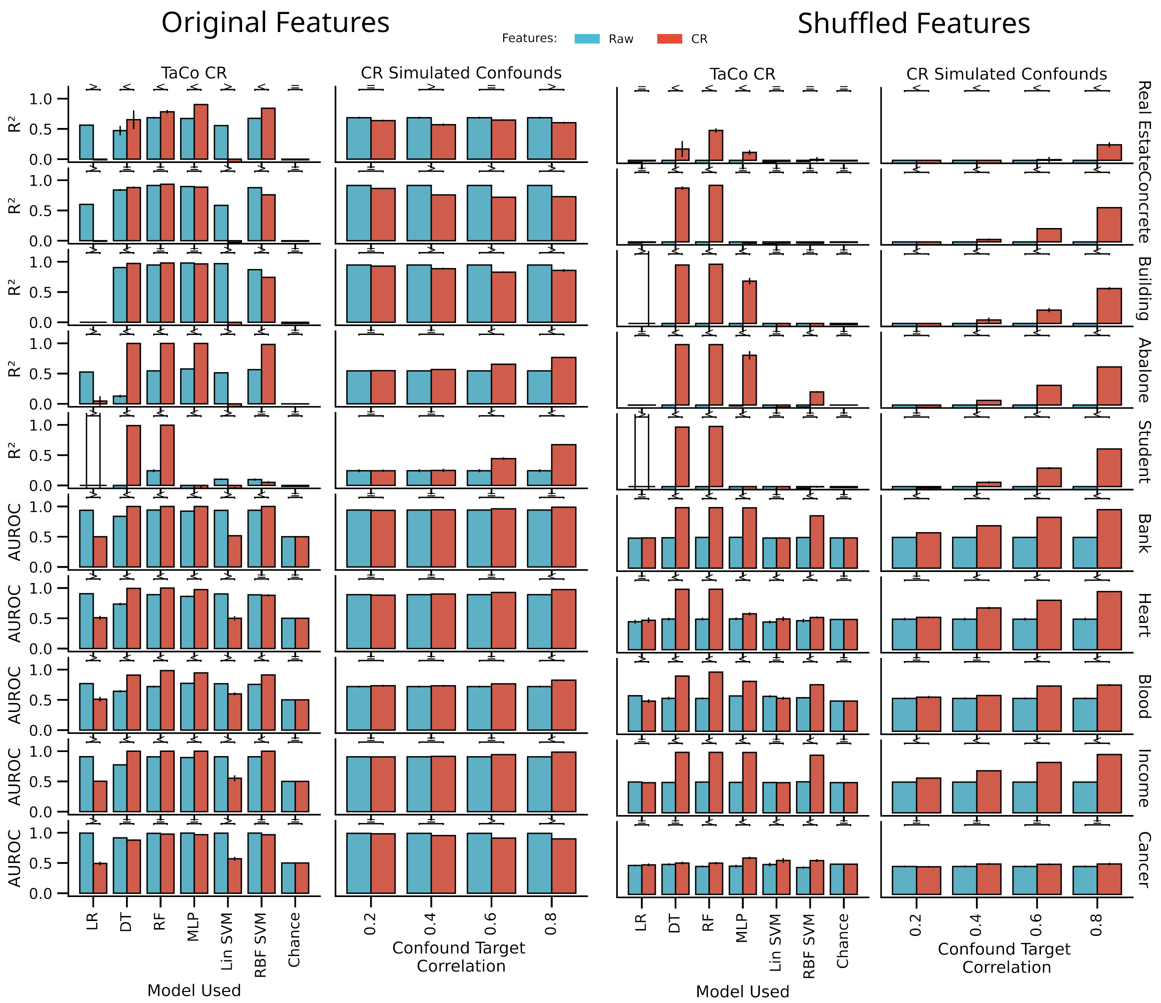

Fig. S1. Performance on the UCI benchmark datasets when using raw vs CR features (a) and raw vs the predicted features given the confound/TaCo/$\hat{X}$ (b). The two columns correspond to: 1) TaCo removal with six ML algorithms (LR, DT, RF, MLP, Lin SVM, RBF SVM), and 2) CR with simulated confound with different correlation to the target (range 0.2-0.8) with RF. (a,b) show performance using the original features while (c,d) show the performance on shuffled features. When using a linear model (LR) TaCo removal leads to reduction in prediction performance, as expected. In contrast, nonlinear models lead to a higher performance for all datasets. This increase could be either explained by confound removal revealing information already in the data (suppression) or confound removal leaking information into the features (confound-leakage). Shuffling the features destroys association between features and the target, therefore subsequent performance increase after TaCo removal indicates the possibility of confound-leakage (c,d). The simulated confounds show that an increase after CR is also possible for confounds weakly related to the target (b,d) and one dataset (Blood) shows strong evidence of confound-leakage.

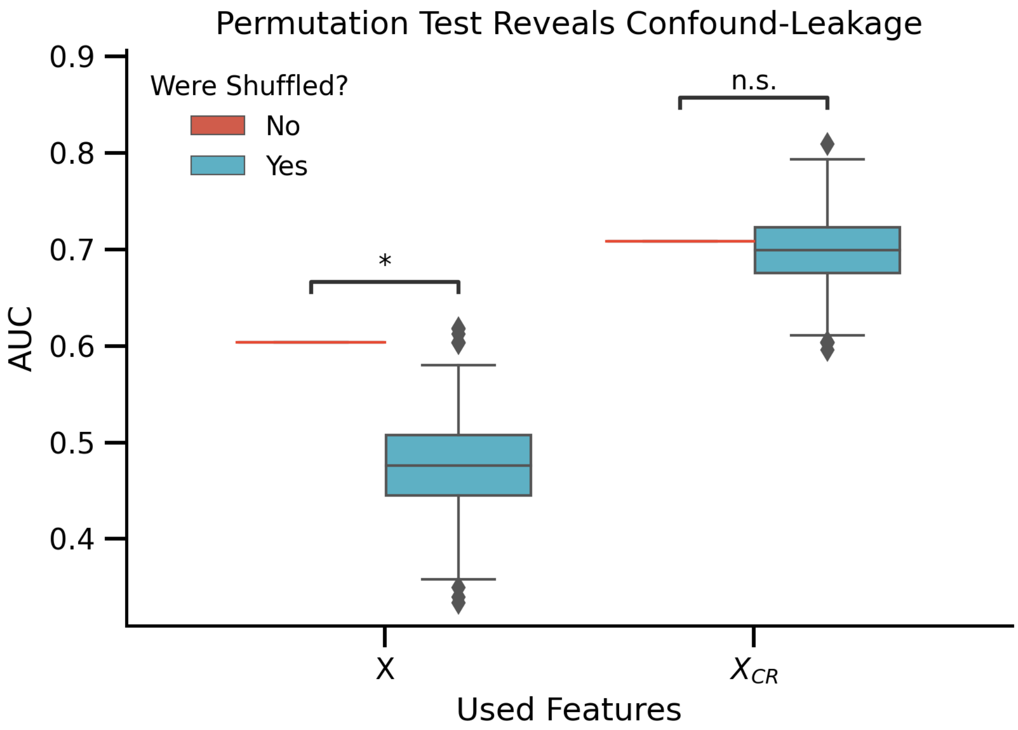

Fig. S2. We performed permutation testing with 1000 iterations. After shuffling the features, a significantly lower performance was observed compared to the original features X. No significant difference between raw and shuffled features was observed when using the X_{CR} features. This result is in line with the leakage hypothesis as the higher accuracy after shuffling and CR indicates leaking target-related confounding information into the features.
