# Supplementary material for: Confound-leakage: confound removal in machine learning leads to leakage
Source: Gigascience. 2023 Sep 30;12:giad071. doi: 10.1093/gigascience/giad071 (PMC10541796; doi:10.1093/gigascience/giad071)
Supplement: giad071_Response_to_Reviewer_Comments_Original_Submission [file giad071_response_to_reviewer_comments_original_submission.pdf]

Author's Response To Reviewer Comments

The point-by-point response was uploaded as a "Personal cover" PDF file.
Please note that there are two "Personal cover" files, one the actual cover letter and the other contains response to reviewers' comments.

We thank the reviewers for their insightful and detailed comments and the oppourtunity to revise our manuscript.
We have addressed all the comments and belive that the manuscript has improved considerably and the new analyses support the main finding of "confound leakage".
