# Supplementary material for: Confound-leakage: confound removal in machine learning leads to leakage
Source: Gigascience. 2023 Sep 30;12:giad071. doi: 10.1093/gigascience/giad071 (PMC10541796; doi:10.1093/gigascience/giad071)
Supplement: giad071_Response_to_Reviewer_Comments_Revision_1 [file giad071_response_to_reviewer_comments_revision_1.pdf]

Author's Response To Reviewer Comments

1) Please add a citation to your new GigaDB data set in the reference list, including the doi link. The citation is:

[xx] Hamdan S, Love BC, von Polier GG, Weis S, Schwender H, Eickhoff SB, Patil KR Supporting data for " Confound-leakage: Confound Removal in Machine Learning Leads to Leakage" GigaScience Database. 2023 http://dx.doi.org/10.5524/102420

In the "availability" section, please write something along the lines "An archival copy of the code and supporting data is also available via the GigaScience database, GigaDB [xx]".

On a minor note, the section currently named "Availability of supporting data and materials" should just be called "Data Availability". (and there are a few typos in this section - please correct).

Response: We have addressed those comments.

2) Please upload editable source files (incl bibliography and style files etc) to Editorial Manager (if the manuscript doesn't convert in EM, you can upload a PDF and include the source files as "supporting material" - but please double check that the source files and pdf are identical)

Response: We have uploaded the source files as well as the compiled PDF.
