# Supplementary material for: Confound-leakage: confound removal in machine learning leads to leakage
Source: Gigascience. 2023 Sep 30;12:giad071. doi: 10.1093/gigascience/giad071 (PMC10541796; doi:10.1093/gigascience/giad071)
Supplement: giad071_Response_to_Reviewer_Comments_Revision_2 [file giad071_response_to_reviewer_comments_revision_2.pdf]

Author's Response To Reviewer Comments

We have updated the manuscript as requested.
