# Supplementary material for: Confound-leakage: confound removal in machine learning leads to leakage
Source: Gigascience. 2023 Sep 30;12:giad071. doi: 10.1093/gigascience/giad071 (PMC10541796; doi:10.1093/gigascience/giad071)
Supplement: giad071_Reviewer_1_Report_Original_Submission — Richard Dinga -- 2/8/2023 Reviewed [file giad071_reviewer_1_report_original_submission.pdf]

Reviewer Report

Title: Confound-leakage: Confound Removal in Machine Learning Leads to Leakage

Version: Original Submission **Date:** 2/8/2023

Reviewer name: Richard Dinga

Reviewer Comments to Author:

The authors wrote a paper about the dangers of feature-wise linear confound removal for nonlinear machine learning, specifically that it can lead to spuriously increased ML performance, what they call "leakage." I believe that the phenomenon they described is real, dangerous, and other researchers or ML practitioners should be aware of it and try to avoid it, and to the best of my knowledge, it was not yet been described in the literature. I even think that what the authors describe also applies to linear models, making it more impactful.

I think there is a missing piece of the explanation of this phenomenon that should be added. Also, pitfalls of confound regression and a possible method for dealing with it were discussed extensively in our paper [1] Dinga et al., "Controlling for Effects of Confounding Variables on Machine Learning Predictions." bioRxiv, 2020. https://doi.org/10.1101/2020.08.17.255034 and the connection should be discussed more.

1. Confound leakage due to implementation of performance measures: One of the main pieces of evidence for the "confound leakage" in the paper is that after shuffling features that there should be no relationship between features and the outcome, but after applying confound regression (CR), ML models can predict the outcome with above-chance performance. In my opinion, the explanation authors provide makes sense, and the data do produce this behavior. However, This is a tricky problem, and I might be wrong here, but I think that this bias only happens because performance measures implemented in scikit-learn (and other libraries, I am sure as well) always report above-chance performance, so AUC will never be < 0.5 and R2 < 0 (i.e., if model predictions would result to AUC = 0.98 or 0.02, scikit-learn will in both cases report 0.98). The bias, as described in the paper, does happen, but half the time, it's positive and half the time negative, and in the long run, it will average to 0. (See also my next point).

To elaborate, after feature shuffling, there should be no relationship between the features and outcome or features and confounds, so there should be no way to learn something in the training set that would translate to above-chance predictions on the test set. Confound removal will create a confound-dependent bias and hence a spurious signal in training set that a model will learn. However, in the test set, this bias will randomly half the time be in the opposite direction. So in the test set, the confound removal induced bias will half the time lead to significantly spuriously above chance and half the time below chance prediction performance. This does not mean that using different AUC implementations would solve the problem or that the problem is less severe, as described in the paper. Results in each test set will still be biased (positively or negatively), with possibly severe consequences for the validity of the results. This should be explored and explained in the paper, and the recommendations should be changed accordingly.

2. Confound regression in linear models: I think the problems of CR, as described in this paper, will also be present in linear models, thus making the findings more impactful, and it should be added to the paper. Here is an example with categorical data, if you excuse R code:
```r
set.seed(1)
n <- 1000
df <- data.frame("x" = rep(c(0,1), n),
"y" = rbinom(n, 1, 0.25))
df$x_cr <- resid(lm(x~y, data=df))
library(MLmetrics)
MLmetrics::AUC(df$x_cr, df$y)
MLmetrics::AUC(df$x, df$y)
```
Here I do not fit any model, and I do not bother with the test set, but the AUC of a linear model fitted on x or x_cr (feature x, after the confound removal) will be the same as the AUC of x or x_cr variables themself. Also, notice that with different seeds, AUC will sometimes be 0.25 and sometimes 0.75, although scikit-learn would report 0.75, following the previous point.

Basically, the confound regression applied to a binary variable will shift the variable slightly with respect to the confound/target, thus increasing the number of unique values of the variable and creating a correlation between the variable and the outcome. Since the shift is random, the correlation will also be random, sometimes positive and sometimes negative. Also, in [1], we describe situations where linear models can learn confounding information from the data after confound cleaning, especially robust linear models, and I suspect similar issues can also lead to biases described in this manuscript.

3. Permutation test: The biases caused by confound regression (positive and negative, or just positive) should also be presented in the permutation distribution, so using a permutation test will not change the bias of the results, but the results will at least not be statistically significant, so it can guard in some way against this problem. Also, an inspection of the permutation distribution might warn researchers about possible problems as described in the paper, e.g., if the distribution is not centered at 0 or is bimodal, it might suggest the "confound leakage." Although the permutation test is not always computationally feasible, I think it should be discussed in the paper. Another way how a permutation test can help is by using stratified shuffling, where shuffling is performed only within confound categories.

4. Analysis of confound predicted features: I do not understand the usage of features predicted by the confound as an analysis step. As far as I understand it, these are just a deterministic function of the confound, so I do not see any additional information that could be obtained by examining the confounded predicted features and their relationship to the outcome instead of confounds themself.

5. References: I think that our paper [1] should be referred to more extensively. We did not describe the problems described in this manuscript, but our findings are relevant to his paper and will also interest readers. Specifically:

a. We have extensively discussed the pitfalls of confound removal. This should be mentioned in the introduction and discussion when discussing the confound removal. Consider discussing these pitfalls in a few sentences. In light of our paper, instead of framing confound regression as something that is

expected to work, it should be framed as something that was already reported to be problematic.
b. The explanations in the section "confound-leakage due to deviation from normal distributions," including figures 1e and 1f, is related to our explanations of why confound removal does not sufficiently clean data from confounding information, and I think this should be mentioned.
c. Using a target as a confound to test the validity/reliability of confound removal was also done in our paper, and I think it should be mentioned.
d. The method to deal with confounding presented in our paper will guard against the dangers of confound regression described in this manuscript. Our method is based on estimating what variance in the outcome can be explained using ML predictions that confounds cannot explain, so in the target as confound situation, this will be 0, thus the dangers of confounded results should be avoided. This should be discussed more in the recommendation section. Also, statements like "we made progress on understanding these issues, there is no full-proof method for detecting and eliminating leakage," and "we are not aware of a procedure to definitively exclude confound-leakage as an explanation." etc. should be modified since I believe that our method, although not perfect, is a candidate solution for this problem and it would of interest to readers to offer them a solution, of course with caveats. Another solution not discussed in the manuscript would be the permutation test mentioned in point 3.
6. High-dimensional data are more susceptible to confound leakage: I am not entirely sure this claim is correct. For me, intuitively, it is not. I can imagine that biases created within individual features by confound removal might average out to 0 in high-dimensional data in the test set. I think it would be worth performing a controlled simulation to support this claim.
Minor
7. I think the methods used need a little more explanation, and the structure could be a little improved. Although what "target as confound" does is, in a way, self-evident, the procedure and motivation for it can be explained a bit more. Also, now the whole explanation is at the end of the introduction section, but it should be elsewhere.
8. possible bug: I suspect that in your walkthrough analysis, X_train_shuffled variable is not shuffled since you are calling X_train_shuffled = pd.DataFrame(zscore.transform(X_train), columns=col_names[:-1])
and not
X_train_shuffled = pd.DataFrame(np.random.permutation(zscore.transform(X_train)), columns=col_names[:-1])
as in other variables. But I am not an expert on the workings of the code. Nevertheless, it won't change anything about the paper. The biases described by the authors will still be present.
Richard Dinga

Methods

Are the methods appropriate to the aims of the study, are they well described, and are necessary controls included? Choose an item.

Conclusions

Are the conclusions adequately supported by the data shown? Choose an item.

Reporting Standards

Does the manuscript adhere to the journal's guidelines on minimum standards of reporting? Choose an item.

Choose an item.

Statistics

Are you able to assess all statistics in the manuscript, including the appropriateness of statistical tests used? Choose an item.

Quality of Written English

Please indicate the quality of language in the manuscript: Choose an item.

Declaration of Competing Interests

Please complete a declaration of competing interests, considering the following questions:

- Have you in the past five years received reimbursements, fees, funding, or salary from an organisation that may in any way gain or lose financially from the publication of this manuscript, either now or in the future?
- Do you hold any stocks or shares in an organisation that may in any way gain or lose financially from the publication of this manuscript, either now or in the future?
- Do you hold or are you currently applying for any patents relating to the content of the manuscript?
- Have you received reimbursements, fees, funding, or salary from an organization that holds or has applied for patents relating to the content of the manuscript?
- Do you have any other financial competing interests?
- Do you have any non-financial competing interests in relation to this paper?

If you can answer no to all of the above, write 'I declare that I have no competing interests' below. If your reply is yes to any, please give details below.

I declare that I have no competing interests

I agree to the open peer review policy of the journal. I understand that my name will be included on my report to the authors and, if the manuscript is accepted for publication, my named report including any attachments I upload will be posted on the website along with the authors' responses. I agree for my report to be made available under an Open Access Creative Commons CC-BY license (http://creativecommons.org/licenses/by/4.0/). I understand that any comments which I do not wish to be included in my named report can be included as confidential comments to the editors, which will not be published.

Choose an item.

To further support our reviewers, we have joined with Publons, where you can gain additional credit to further highlight your hard work (see: https://publons.com/journal/530/gigascience). On publication of this paper, your review will be automatically added to Publons, you can then choose whether or not to claim your Publons credit. I understand this statement.

Yes Choose an item.
