# Supplementary material for: Confound-leakage: confound removal in machine learning leads to leakage
Source: Gigascience. 2023 Sep 30;12:giad071. doi: 10.1093/gigascience/giad071 (PMC10541796; doi:10.1093/gigascience/giad071)
Supplement: giad071_Reviewer_2_Report_Original_Submission — Qingyu Zhao -- 2/15/2023 Reviewed [file giad071_reviewer_2_report_original_submission.pdf]

Reviewer Report

Title: Confound-leakage: Confound Removal in Machine Learning Leads to Leakage

Version: Original Submission **Date:** 2/15/2023

Reviewer name: Qingyu Zhao

Reviewer Comments to Author:

The manuscript overrides the common perception that linear regression can help remove confounding effect in machine learning analysis. Experiments showed that in certain scenarios linear regression can inject confounding effects that can be picked up by non-linear machine learning models, e.g., by achieving higher prediction accuracy even when using shuffled features. Two hypotheses are given why such phenomenon can happen.
I think some part of the analysis resolved my long-lasting confusion in my own studies. Interesting observations overall, but there are three major concerns that I hope the authors can respond to:
1. While linear regression is a standard way to remove confounding effects, how to construct that regression can vary from case to case. In the scenario where the confounder is highly correlated with the label, people sometimes incorporate both label and confounder as covariates, or apply linear regression only in the control group. The idea is that you need to disentangle variance in the feature explained by the label from the variance explained by the confound. Again, feature, label, and confound have three-way dependencies by definition and failure to discuss this aspect can be misleading.
2. I generally do not agree with the claim that "\hat{X} can only be at most as predictive as X", especially in the TaCo setting. Using label as the super confound, we are basically trying to explicitly preserve label-related component in the feature and discard information irrelevant to the label. This actually makes the classification simpler, which does not indicate confounder-leakage. If authors agree with my view, I would suggest changing their interpretation of this part of the results or removing them.
3. Most of the analyses were based on simulation. Most of the interpretation was based on accuracy scores. What is more interesting is the mechanism why it happens in real applications. The authors have two hypotheses, which I believe are not that hard to test on the real data set. One can look at features that drive the X_CR classification but not the raw classification, and then look at how their distribution changes with respect to confounders. One can probably visualize the distribution shift over multi-dimensional features using certain dimension reduction techniques. With those results, the manuscript will become more valid.
Minor: figure captions are generally terse. Axis labels are confusing, e.g., Figure 1c,d. In figure 2, why are there two rows of r2 and two rows of AUC? What do you mean by those four rows of 'score'? Do they belong to the right column? Do < and > indicate statistical significance? People often use *, ** to indicate p<0.05, p<0.005, because the direction is obvious from the plots already.

Methods

Are the methods appropriate to the aims of the study, are they well described, and are necessary controls included? Choose an item.

Conclusions

Are the conclusions adequately supported by the data shown? Choose an item.

Reporting Standards

Does the manuscript adhere to the journal's guidelines on minimum standards of reporting? Choose an item.

Choose an item.

Statistics

Are you able to assess all statistics in the manuscript, including the appropriateness of statistical tests used? Choose an item.

Quality of Written English

Please indicate the quality of language in the manuscript: Choose an item.

Declaration of Competing Interests

Please complete a declaration of competing interests, considering the following questions:

- Have you in the past five years received reimbursements, fees, funding, or salary from an organisation that may in any way gain or lose financially from the publication of this manuscript, either now or in the future?
- Do you hold any stocks or shares in an organisation that may in any way gain or lose financially from the publication of this manuscript, either now or in the future?
- Do you hold or are you currently applying for any patents relating to the content of the manuscript?
- Have you received reimbursements, fees, funding, or salary from an organization that holds or has applied for patents relating to the content of the manuscript?
- Do you have any other financial competing interests?
- Do you have any non-financial competing interests in relation to this paper?

If you can answer no to all of the above, write 'I declare that I have no competing interests' below. If your reply is yes to any, please give details below.

no competing interest

I agree to the open peer review policy of the journal. I understand that my name will be included on my report to the authors and, if the manuscript is accepted for publication, my named report including any attachments I upload will be posted on the website along with the authors' responses. I agree for my

report to be made available under an Open Access Creative Commons CC-BY license (http://creativecommons.org/licenses/by/4.0/). I understand that any comments which I do not wish to be included in my named report can be included as confidential comments to the editors, which will not be published.

Choose an item.

To further support our reviewers, we have joined with Publons, where you can gain additional credit to further highlight your hard work (see: https://publons.com/journal/530/gigascience). On publication of this paper, your review will be automatically added to Publons, you can then choose whether or not to claim your Publons credit. I understand this statement.

Yes Choose an item.
